# A geometric method for eigenvalue problems with low-rank perturbations

**DOI:** 10.1098/rsos.170390

**Published:** 2017-09-27

**Authors:** Thomas J. Anastasio, Andrea K. Barreiro, Jared C. Bronski

**Affiliations:** 1 Department of Molecular and Integrative Physiology and Beckman Institute, University of Illinois Urbana-Champaign, Urbana, IL 61820, USA; 2 Department of Mathematics, Southern Methodist University, PO Box 750156, Dallas, TX 75275, USA; 3 Department of Mathematics, University of Illinois Urbana-Champaign, 1409 West Green Street, Urbana, IL 61801, USA

**Keywords:** bifurcation theory, Aronszajn–Krein formula, rank-one perturbations

## Abstract

We consider the problem of finding the spectrum of an operator taking the form of a low-rank (rank one or two) non-normal perturbation of a well-understood operator, motivated by a number of problems of applied interest which take this form. We use the fact that the system is a low-rank perturbation of a solved problem, together with a simple idea of classical differential geometry (the envelope of a family of curves) to completely analyse the spectrum. We use these techniques to analyse three problems of this form: a model of the oculomotor integrator due to Anastasio & Gad (2007 *J. Comput. Neurosci.* **22**, 239–254. (doi:10.1007/s10827-006-0010-x)), a continuum integrator model, and a non-local model of phase separation due to Rubinstein & Sternberg (1992 *IMA J. Appl. Math.* **48**, 249–264. (doi:10.1093/imamat/48.3.249)).

## 1. Introduction

In this paper, we analyse eigenvalue problems of the following form:

1.1 $$\tilde{\text{M}}w=\text{M}w+\rho_{1}f_{1}\langle g_{1},w\rangle +\rho_{2}f_{2}\langle g_{2},w\rangle =\lambda w,$$

with *ρ*_1_ and *ρ*_2_ parameters, ***f***_*i*_,***g***_*i*_ fixed vectors and **M** is an operator with known spectrum. While equation (1.1) might appear very specific, we are aware of a number of interesting eigenvalue problems which take this form. These include:

— A model due to Anastasio and Gad of the behaviour of the oculomotor integrator [1].
— A non-local Allen–Cahn model due to Rubinstein and Sternberg for phase separation [2].
— The stability problem for spike solutions to activator–inhibitor models in the limit of slow activator diffusion [3,4].
— Stability for models of runaway ohmic heating [5–7] and microwave heating [8].
— Stability for stationary solutions of model for phytoplankton growth [9].

Four of the models take the form of a reaction–diffusion equation with a non-local term. The study of the stability of stationary solutions of such models naturally leads to an eigenvalue problem which takes the form of a self-adjoint Sturm–Liouville operator plus a finite-rank perturbation coming from the non-local term. Freitas [3] has considered a similar problem and has some related results for a single perturbation (rather than a two parameter family); Bose & Kriegsmann [8] have some other related results, mainly in the case where ***f***_*i*_=***g***_*i*_ where the problem is self-adjoint. We refer the interested reader to the review paper of Freitas [10], which details a number of models whose stability problems take the form of a ‘nice’ operator with a low-rank (typically rank one) perturbation.

In all of these problems, the eigenvalue problem arises in the study of the stability of a particular steady state. In this situation, one is typically interested in understanding qualitative properties of the spectrum as a function of the parameters (*ρ*_1_,*ρ*_2_). In particular, one might wish to understand, for any particular pair (*ρ*_1_,*ρ*_2_),

— how many eigenvalues are in the right half-plane, and
— how many eigenvalues are real (versus complex).

Here we give a direct way to construct a phase-diagram in the (*ρ*_1_,*ρ*_2_) plane which answers these questions.

The main approach that we take here is to exploit the low-rank nature of the perturbations, along with some geometric constructions for the quantities of interest. Since the eigenvalues will vary continuously as a function of the parameters (*ρ*_1_,*ρ*_2_), the quantities noted above are constant on open sets, with the boundary of these sets being, respectively

— the set of (*ρ*_1_,*ρ*_2_) for which $\tilde{\text{M}}$ has a purely complex eigenvalue λ=ı*ω* (including λ=0), and
— the set of (*ρ*_1_,*ρ*_2_) for which $\tilde{\text{M}}$ has a double real eigenvalue.

Knowledge of these boundary sets, together with knowledge of the spectrum of the unperturbed operator **M**, would therefore enable us to study stability in the entire plane.

In passing we note that, while the unperturbed operator **M** is self-adjoint in some of the examples presented here, self-adjointness is not strictly necessary. What *is* necessary is that the spectrum of the unperturbed operator **M** is known (at least qualitatively), so that we have a baseline with which to compare the perturbed operator (much as a constant of integration fixes a particular solution of a differential equation). In each example studied here, the unperturbed operator **M** will have a purely real spectrum.

Our main result in this paper will be to identify—and give a recipe for computing—a set of geometric quantities associated with the spectrum of a rank-two perturbation of a well-known operator, which can be used to analyse the perturbed operator in the entire plane: this is done in §2. We will then apply our technique to three specific problems which can be written in the form of equation (1.1). The first is a model of a coupled brainstem–cerebellum neuronal network called the *oculomotor integrator* (§3); the second is a continuum version of that model, in which the (relatively numerous) brainstem neurons are replaced by a neural ‘line’ (§4). Finally, we analyse a stability problem that arises in a nonlocal reaction–diffusion equation [2] (§5). In this last problem, we also use an intermediate result from §2 (the Aronszajn–Krein formula and its consequences) to prove a new theorem about stability of stationary solutions.

## 2. Basic calculations

We begin with some general dimension-counting arguments. The matrix^1^ $\tilde{\text{M}}$ is assumed to be a real *N*×*N* matrix. Real non-symmetric matrices will generically have a real eigenvalue of multiplicity higher than one on a set of codimension one. In a two-parameter model such as we are considering here this codimension one set divides the parameter space into open sets having a constant number of real eigenvalues. As one crosses this set the number of real eigenvalues changes by (generically) two. This motivates the following definition:

**Definition 2.1**

We define the bifurcation curve to be the locus of points $V=(\rho_{1},\rho_{2})$ for which $\tilde{\text{M}}$ has a real eigenvalue of multiplicity two or higher.

For matrix problems, of course, there exists an algebraic procedure for determining the values in the (*ρ*_1_,*ρ*_2_) plane where the matrix has multiple eigenvalues. One can simply compute the discriminant (in λ) of the characteristic polynomial of the matrix $\tilde{\text{M}},$

$${\mathrm{disc}}_{\lambda }(\det(\tilde{\text{M}}-\lambda \text{I}))=P(\rho_{1},\rho_{2}),$$

which gives a polynomial in the parameters (*ρ*_1_,*ρ*_2_). The variety defined by the zero set of this polynomial

$$V=\{(\rho_{1},\rho_{2})|P(\rho_{1},\rho_{2})=0\},$$

determines the bifurcation curve. Unfortunately, this computation is not practical to carry out analytically for real problems: for a large matrix, *P*(*ρ*_1_,*ρ*_2_) will be a polynomial of large degree and the zero set will be difficult to compute. For the case of operators, even very nice ones, it is not clear that the discriminant makes sense at all.

Instead we use the fact that the perturbations are of finite rank to give an explicit rational or algebraic parametrization of the bifurcation curve. We begin by stating a preliminary lemma, which is basically the Aronszajn–Krein formula for rank-one perturbations:

**Lemma 2.2**

*Let* $\tilde{M}$ *be an N*×*N matrix defined as in equation (1.1). The characteristic polynomial of* $\tilde{M}$

$$\tilde{D}(\lambda )=\det(\tilde{M}-\lambda I),$$

*takes the following form*:

2.1 $$\det(\tilde{M}-\lambda I)=D(\lambda )+P_{1}(\lambda )\rho_{1}+P_{2}(\lambda )\rho_{2}+Q(\lambda )\rho_{1}\rho_{2},$$

*where* D(λ)=det(M−λI) *is the determinant of the unperturbed problem, P_*i*_*(λ) *are polynomials of degree (at most) (N*−1) *and Q*(λ) *of degree (at most) N*−2. *In the case where* ***g***_1_ *and* ***g***_2_ (*or* ***f***_1,2_) *are linearly dependent* *Q*(λ)=0.

**Proof.**

Owing to the rank-two nature of the perturbation, the characteristic polynomial can contain no powers of *ρ*_1_ or *ρ*_2_ above the first. The easiest way to see this is via multilinear algebra. The determinant is clearly polynomial in λ,*ρ*_1_,*ρ*_2_. A general term of the form $\rho_{1}^{j}\rho_{2}^{k}$ comes from the wedge product of *j* factors of ***g***_1_, *k* factors of ***g***_2_ and (*N*−(*j*+*k*)) columns from **M**−λ**I**. Any term with more than one factor of ***g***_1_ and one factor of ***g***_2_ must be zero. Hence the determinant is of the form given in equation (2.1).

Finally, if ***g***_1_ and ***g***_2_ are linearly dependent, then any wedge product including both ***g***_1_ and ***g***_2_ will vanish, so that *Q*(λ)≡0.

The explicit form of the polynomials *P*_*i*_(λ),*Q*(λ) is easy to compute from the above construction (a detailed derivation is provided in appendix A). If we let cof^t^(**M**−λ**I**) denote the transpose cofactor matrix of **M**−λ**I**, then one has the following formulae:

2.2 $$\begin{array}{rl} P_{1}(\lambda ) & =\langle g_{1},{\mathrm{cof}}^{t}(\text{M}-\lambda \text{I})f_{1}\rangle ,\\ P_{2}(\lambda ) & =\langle g_{2},{\mathrm{cof}}^{t}(\text{M}-\lambda \text{I})f_{2}\rangle \\ \;\text{and}\;Q(\lambda ) & =\frac{1}{\det(\text{M}-\lambda \text{I})}\left|\begin{array}{cc} \left\langle g_{1},{\mathrm{cof}}^{t}(\text{M}-\lambda \text{I})f_{1}\right\rangle & \left\langle g_{1},{\mathrm{cof}}^{t}(\text{M}-\lambda \text{I})f_{2}\right\rangle \\ \left\langle g_{2},{\mathrm{cof}}^{t}(\text{M}-\lambda \text{I})f_{1}\right\rangle & \left\langle g_{2},{\mathrm{cof}}^{t}(\text{M}-\lambda \text{I})f_{2}\right\rangle \end{array}\right|. \end{array}\}$$

If in addition **M** is self-adjoint, we have alternative formulae, in which the polynomial form of *Q*(λ), etc., is even more evident:

2.3 $$\begin{array}{rl} P_{1}(\lambda ) & =\sum_{i}\langle \;f_{1},\phi_{i}\rangle \langle g_{1},\phi_{i}\rangle \prod_{j\neq i}(\lambda_{j}-\lambda )\\ P_{2}(\lambda ) & =\sum_{i}\langle \;f_{2},\phi_{i}\rangle \langle g_{2},\phi_{i}\rangle \prod_{j\neq i}(\lambda_{j}-\lambda )\\ \;\text{and}\;Q(\lambda ) & =\sum_{i,j}(\langle \;f_{1},\phi_{i}\rangle \langle g_{1},\phi_{i}\rangle \langle \;f_{2},\phi_{j}\rangle \langle g_{2},\phi_{j}\rangle \\ & \;-\langle \;f_{1},\phi_{i}\rangle \langle g_{2},\phi_{i}\rangle \langle \;f_{2},\phi_{j}\rangle \langle g_{1},\phi_{j}\rangle )\prod_{k\neq i,j}(\lambda_{k}-\lambda ), \end{array}\}$$

where λ_*i*_ and ***ϕ***_*i*_ are the eigenvalues and eigenvectors of the unperturbed matrix **M**. ▪

It is often more convenient to divide equation (2.1) by det(M−λI) to put the eigenvalue condition in the form

2.4 $$\begin{array}{rl} 0 & =1+\rho_{1}\langle g_{1},{\left(\text{M}-\lambda \text{I}\right)}^{-1}f_{1}\rangle +\rho_{2}\langle g_{2},{\left(\text{M}-\lambda \text{I}\right)}^{-1}f_{2}\rangle +\rho_{1}\rho_{2}(\langle g_{1},{\left(\text{M}-\lambda \text{I}\right)}^{-1}f_{1}\rangle \langle g_{2},{\left(\text{M}-\lambda \text{I}\right)}^{-1}f_{2}\rangle \\ & \;-\langle g_{1},{\left(\text{M}-\lambda \text{I}\right)}^{-1}f_{2}\rangle \langle g_{2},{\left(\text{M}-\lambda \text{I}\right)}^{-1}f_{1}\rangle ); \end{array}$$

or even more simply, when *Q*(λ)=0,

$$0=1+\rho_{1}\langle \;g_{1},{\left(\text{M}-\lambda \text{I}\right)}^{-1}f_{1}\rangle +\rho_{2}\langle g_{2},{\left(\text{M}-\lambda \text{I}\right)}^{-1}f_{2}\rangle .$$

This form has the advantage that it is expressed in terms of resolvents (i.e. *R*_λ_≡(**M**−λ**I**)^−1^), which are defined very generally for operators, rather than determinants and cofactors, which are not.

One geometric way to interpret this characteristic polynomial is as defining a one-parameter family of rational curves, the curves of constant eigenvalue. For each value of λ, equation (2.1) defines a curve in the (*ρ*_1_,*ρ*_2_) plane along which λ is an eigenvalue. For instance, the matrix has zero as an eigenvalue along the curve

$$\begin{array}{rl} & D(0)+\rho_{1}P_{1}(0)+\rho_{2}P_{2}(0)+\rho_{1}\rho_{2}Q(0)=0\\ & \;\Rightarrow \rho_{2}=-\frac{D(0)+\rho_{1}P_{1}(0)}{P_{2}(0)+\rho_{1}Q(0)} \end{array}$$

in the (*ρ*_1_,*ρ*_2_) plane. In the special case where *Q*(λ)≡0, equation (2.1) defines a one-parameter family of lines.

Given a family of curves, it is always fruitful to consider its *envelope*, which is the simultaneous solution to

2.5 $$D(\lambda )+\rho_{1}P_{1}(\lambda )+\rho_{2}P_{2}(\lambda )+\rho_{1}\rho_{2}Q(\lambda )=0$$

and

2.6 $$D^{\prime }(\lambda )+\rho_{1}P_{1}^{\prime }(\lambda )+\rho_{2}P_{2}^{\prime }(\lambda )+\rho_{1}\rho_{2}Q^{\prime }(\lambda )=0.$$

Whereas each curve in the family encodes information on the location of a particular eigenvalue, the envelope of the curve encodes information on eigenvalue coincidence: this is the content of the next lemma. To simplify notation, we use the wedge notation for Wronskians: *f*∧*g*=*fg*′−*f*′*g*.

**Lemma 2.3**

*The solutions to* (2.5), (2.6) *give the bifurcation curve* V. *If* *Q*(λ) *is not identically zero the bifurcation curve* V *is generically given by the union of the pair of parametric curves given by*

2.7 $$\rho_{1}=\frac{-(P_{1}\wedge P_{2}+D\wedge Q)\pm \sqrt{{\left(P_{1}\wedge P_{2}-D\wedge Q\right)}^{2}-4(D\wedge P_{1})(P_{2}\wedge Q)}}{2(P_{1}\wedge Q)}$$

*and*

2.8 $$\rho_{2}=\frac{P_{1}\wedge P_{2}-D\wedge Q\mp \sqrt{{\left(P_{1}\wedge P_{2}-D\wedge Q\right)}^{2}-4(D\wedge P_{1})(P_{2}\wedge Q)}}{2(P_{2}\wedge Q)}.$$

*If* *Q*(λ) *is identically zero, then the bifurcation curve is generically given by the parametric curve*

2.9 $$\rho_{1}=-\frac{P_{2}\wedge D(\lambda )}{P_{1}\wedge P_{2}(\lambda )}$$

*and*

2.10 $$\rho_{2}=\frac{P_{1}\wedge D(\lambda )}{P_{1}\wedge P_{2}(\lambda )}.$$

**Proof.**

The equivalence of the envelope and the discriminant is standard—see, for instance, Bruce & Giblin [11] or Spivak [12]. Generically, one just has to solve equations (2.5) and (2.6) for *ρ*_1_ and *ρ*_2_, respectively. This is equivalent to solving a linear and a quadratic equation in the general case and a pair of linear equations in the special case *Q*(λ)=0.

For certain values of λ the system (2.5), (2.6) maybe be inconsistent, or consistent but underdetermined. Inconsistency indicates that this eigenvalue cannot be achieved by any choice of *ρ*_1,2_; this will occur if

$$\mathrm{rank}\left(\begin{array}{ccc} P_{1} & P_{2} & Q\\ P_{1}^{\prime } & P_{2}^{\prime } & Q^{\prime } \end{array}\right)<\mathrm{rank}\left(\begin{array}{cccc} D & P_{1} & P_{2} & Q\\ D^{\prime } & P_{1}^{\prime } & P_{2}^{\prime } & Q^{\prime } \end{array}\right).$$

If the system is consistent but underdetermined, then there may be a curve in the (*ρ*_1_,*ρ*_2_) plane along which λ is a multiple eigenvalue. The system (2.5), (2.6) is consistent and underdetermined for λ if one of the following conditions **C1**,**C2**,**C3** hold:

2.11 $$\begin{array}{rl} & \text{C1}\;\mathrm{rank}\left(\begin{array}{cccc} D & P_{1} & P_{2} & Q\\ D^{\prime } & P_{1}^{\prime } & P_{2}^{\prime } & Q^{\prime } \end{array}\right)<2, \end{array}$$

2.12 $$\begin{array}{rl} & \text{C2}\left\{ \begin{array}{l} P_{1}\wedge Q(\lambda )\neq 0\\ P_{2}\wedge Q(\lambda )=0\\ D\wedge P_{1}(\lambda )=0\\ P_{1}\wedge P_{2}(\lambda )=D\wedge Q(\lambda ) \end{array}\right. \end{array}$$

2.13 $$\begin{array}{rl} \;\text{and}\; & \text{C3}\left\{ \begin{array}{l} P_{2}\wedge Q(\lambda )\neq 0\\ P_{1}\wedge Q(\lambda )=0\\ D\wedge P_{2}(\lambda )=0\\ P_{2}\wedge P_{1}(\lambda )=D\wedge Q(\lambda ) \end{array}\right. \end{array}$$

If any of these genericity conditions are satisfied for some value of λ, then (generically) there exists a curve in the (*ρ*_1_,*ρ*_2_) plane along which that value of λ is a multiple eigenvalue. If they are not satisfied for any λ, then the bifurcation curve is equal to the envelope curve. Note that one can always check whether or not a pair of polynomials have a common root by computing the resultant of the polynomials: one need *not* be able to explicitly factor the polynomials to test this condition. Thus, the genericity condition is readily checkable. ▪

**Lemma 2.4**

*As* (*ρ*_1_,*ρ*_2_) *are varied so as to cross the envelope the number of real eigenvalues generically changes by two*.

**Proof.**

We assume that most readers are familiar with this phenomenon from the theory of first-order quasi-linear partial differential equations, where the characteristic curves form a one-parameter family of curves and the envelope (caustic) marks the transition between regions which are (typically) singly and triply covered by characteristics, but we give a short proof.

The envelope curve is defined by the simultaneous solution to

$$F(\rho_{1},\rho_{2},\lambda )=0\;\mathrm{and}\;\frac{\partial F}{\partial \lambda }(\rho_{1},\rho_{2},\lambda )=0.$$

Assume that $\left(\rho_{1}^{*},\rho_{2}^{*}\right)$ is a point along this curve with corresponding eigenvalue λ*, and that the gradient $\nabla_{\rho_{1},\rho_{2}}F(\rho_{1}^{*},\rho_{2}^{*},\lambda^{*})$ and the second derivative $\left(\partial^{2}F/\partial \lambda^{2})(\rho_{1}^{*},\rho_{2}^{*},\lambda^{*}\right)$ are non-vanishing. Expanding in a neighbourhood of this point, i.e. letting $\rho_{1}=\rho_{1}^{*}+\delta \rho_{1},\rho_{2}=\rho_{2}^{*}+\delta \rho_{2}$ and λ=λ*+*δ*λ, we find the normal form

$$\frac{1}{2}\frac{\partial^{2}F}{\partial \lambda^{2}}{\left(\delta \lambda \right)}^{2}+\nabla_{\rho_{1},\rho_{2}}F\cdot (\delta \rho_{1},\delta \rho_{2})=O(\delta \rho \delta \lambda ,\delta \rho^{2},\delta \lambda^{3}).$$

By the Weierstrauss preparation theorem, we can approximate solutions to *F*(*ρ*_1_,*ρ*_2_,λ)=0 in a neighbourhood of the point $\left(\rho_{1}^{*},\rho_{2}^{*}\right)$ by fixing (*δρ*_1_,*δρ*_2_) and solving for *δ*λ; i.e. by solving the quadratic equation

**Figure 1.**
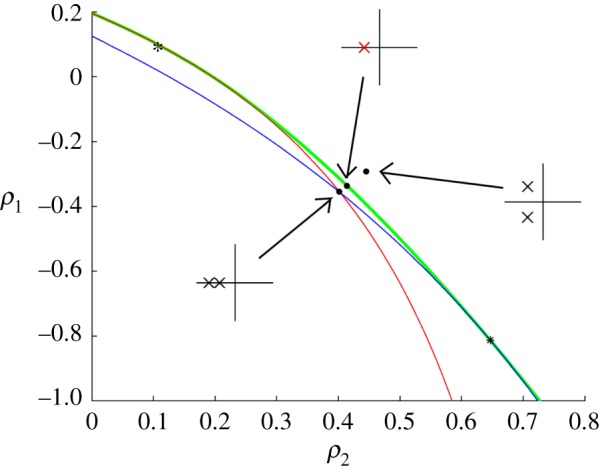
A schematic illustrating the bifurcation of eigenvalues across an envelope curve. The envelope curve (green bold solid) and two constant eigenvalue curves (blue and red light solid) are shown. The inset axes illustrate the relative positions of the eigenvalue pair in the vicinity of the bifurcation point.

2.14 $$\frac{1}{2}\frac{\partial^{2}F}{\partial \lambda^{2}}{\left(\delta \lambda \right)}^{2}+\nabla_{\rho_{1},\rho_{2}}F\cdot (\delta \rho_{1},\delta \rho_{2})=0,$$

for *δ*λ. The tangent line to the envelope at $\left(\rho_{1}^{*},\rho_{2}^{*}\right)$ is given by ∇_*ρ*_1_,*ρ*_2__*F*⋅(*δρ*_1_,*δρ*_2_)=0; on one side of this line, equation (2.14) has two distinct real roots ±*δ*λ, so each point lies on two constant eigenvalue curves, λ*±*δ*λ. On the other side of the tangent line, there are a pair of complex conjugate roots. This general picture is illustrated in figure 1, which shows a close-up of the envelope curve from an example to follow.

For more details on the envelope, see the text of Bruce & Giblin [11]. ▪

We would next like to consider the possibility of eigenvalues of higher multiplicity. The envelope curve is, in general, a well-behaved curve and admits a parametrization by arc length. However, this may fail at an isolated set of points in (*ρ*_1_,*ρ*_2_). The next lemma says that (modulo some genericity assumptions) the following are all equivalent, and occur on a codimension two set (isolated points in the (*ρ*_1_,*ρ*_2_) plane):

— Points in the (*ρ*_1_,*ρ*_2_) plane where the model has a real eigenvalue of multiplicity at least three.
— Points where the tangent vector to the envelope curve vanishes.
— Cusps in the envelope curve.

**Lemma 2.5**

*The vanishing of the tangent vector to the envelope curve at a point implies that* $\tilde{M}$ *has an eigenvalue of multiplicity (at least) three at that point. The converse holds as long as the following determinant is non-zero at the point in question*:

$$\left|\begin{array}{cc} P_{1}+\rho_{2}Q & P_{2}+\rho_{1}Q\\ P_{1}^{\prime }+\rho_{2}Q^{\prime } & P_{2}^{\prime }+\rho_{1}Q^{\prime } \end{array}\right|\neq 0.$$

*Alternatively, the model has a triple eigenvalue if and only if either*

$$\begin{array}{rl} \left(P_{1}\wedge P_{2}\wedge Q)(P_{1}\wedge P_{2}\wedge D\right) & =(D\wedge P_{2}\wedge Q)(P_{1}\wedge D\wedge Q)=0\\ P_{1}\wedge P_{2}\wedge Q & \neq 0 \end{array}$$

*or*

$$P_{1}\wedge P_{2}\wedge Q=P_{1}\wedge P_{2}\wedge D=D\wedge P_{2}\wedge Q=P_{1}\wedge D\wedge Q=0.$$

**Proof.**

The conditions for an eigenvalue of multiplicity (at least) two are given by (2.5) and (2.6). Differentiating each of these with respect to λ gives the following equations for $\rho_{1}^{\prime },\rho_{2}^{\prime }$:

$$\begin{array}{rl} & \rho_{1}^{\prime }(P_{1}(\lambda )+\rho_{2}Q(\lambda ))+\rho_{2}^{\prime }(P_{2}(\lambda )+\rho_{1}Q(\lambda ))=0\\ \;\mathrm{and}\; & \rho_{1}^{\prime }(P_{1}^{\prime }(\lambda )+\rho_{2}Q^{\prime }(\lambda ))+\rho_{2}^{\prime }(P_{2}^{\prime }(\lambda )+\rho_{1}Q^{\prime }(\lambda ))=-(D^{\prime \prime }(\lambda )+\rho_{1}P_{1}^{\prime \prime }(\lambda )+\rho_{2}P_{2}^{\prime \prime }(\lambda )+\rho_{1}\rho_{2}Q^{\prime \prime }). \end{array}$$

The conditions for an eigenvalue of multiplicity at least three are given by (2.5) and (2.6) together with the condition

2.15 $$(D^{\prime \prime }(\lambda )+\rho_{1}P_{1}^{\prime \prime }(\lambda )+\rho_{2}P_{2}^{\prime \prime }(\lambda )+\rho_{1}\rho_{2}Q^{\prime \prime })=0.$$

Thus, it is clear that $\rho_{1}^{\prime }=0,\rho_{2}^{\prime }=0$ implies that the eigenvalue is of multiplicity (at least) three. Further if

$$\left|\begin{array}{cc} P_{1}+\rho_{2}Q & P_{2}+\rho_{1}Q\\ P_{1}^{\prime }+\rho_{2}Q^{\prime } & P_{2}^{\prime }+\rho_{1}Q^{\prime } \end{array}\right|\neq 0,$$

then the above system can be solved uniquely for $\rho_{1}^{\prime },\rho_{2}^{\prime }$ and the existence of an eigenvalue of multiplicity three implies $\rho_{1}^{\prime }=0,\rho_{2}^{\prime }=0.$

A bit more algebra gives another characterization of points where the eigenvalue has multiplicity three or higher. Equations (2.5), (2.6) and (2.15) form a system of three equations in three unknowns *ρ*_1_,*ρ*_2_ and *ρ*_3_=*ρ*_1_*ρ*_2_. Solving these three equations for (*ρ*_1_,*ρ*_2_,*ρ*_3_) and imposing the consistency condition *ρ*_3_=*ρ*_1_*ρ*_2_ shows that one has a root of multiplicity three if and only if either

$$\begin{array}{rl} \left(P_{1}\wedge P_{2}\wedge Q)(P_{1}\wedge P_{2}\wedge D\right) & =(D\wedge P_{2}\wedge Q)(P_{1}\wedge D\wedge Q)\\ P_{1}\wedge P_{2}\wedge Q & \neq 0, \end{array}$$

*or* all of the Wronskians

$$P_{1}\wedge P_{2}\wedge Q=P_{1}\wedge P_{2}\wedge D=D\wedge P_{2}\wedge Q=P_{1}\wedge D\wedge Q=0,$$

vanish. The first possibility is typically of codimension two—it is expected to occur at isolated values of λ corresponding to isolated values of (*ρ*_1_,*ρ*_2_). The second does not typically happen at all, since it requires the simultaneous vanishing of several polynomials. However, in example 2.7, this case occurs because it is forced by a symmetry of the model.

Finally recall that a simple zero of the tangent vector represents a cusp, and generically an eigenvalue of multiplicity at least three will have multiplicity exactly three, so typically cusps in the envelope are equivalent to triple eigenvalues. ▪

The geometry of a bifurcation in the neighbourhood of a triple eigenvalue is illustrated in figure 4*b*. In a neighbourhood of this point, there are three dominant eigenvalues which participate in the bifurcation. The cusp of the envelope represents a transition between a bifurcation between the intermediate and the smallest eigenvalue in the trio, and a bifurcation between the intermediate and the largest eigenvalue in the trio. Emerging from the cusp-point is a curve that represents an exchange of dominance phenomenon, with a complex conjugate pair of eigenvalues crossing a single real one.

When considering questions of stability and the behaviour of the dominant eigenvalue it is also important to understand the behaviour of the complex eigenvalues. In particular, one would like to understand the locus of points at which the matrix has purely imaginary eigenvalues, as this curve indicates where the model loses stability due to a Hopf bifurcation.

**Definition 2.6**

The Hopf curve is the locus of points in the (*ρ*_1_,*ρ*_2_) plane where $\tilde{\text{M}}$ has a pair of purely imaginary eigenvalues. Generically, this curve is given parametrically by

2.16 Re(D(iω))+ρ1Re(P1(iω))+ρ2Re(P2(iω))+ρ1ρ2Re(Q(iω))=0

and

2.17 Im(D(iω))+ρ1Im(P1(iω))+ρ2Im(P2(iω))+ρ1ρ2Im(Q(iω))=0,

where Re, Im represent the real and imaginary parts, respectively. The genericity conditions are the same as in lemma (2.3) with the replacement of the Wronskians *f*∧*g* by the quantities Re( *f*(i*ω*)) Im(*g*(i*ω*))−Re(*g*(i*ω*))Im(*f*(i*ω*)).

The Hopf curve, the envelope, and the λ=0 eigenvalue curve will all intersect at a single point, the point at which there is a zero eigenvalue of higher multiplicity. We now present an illustrative example.

**Example 2.7**

We consider the following model:

$$\text{M}=\left(\begin{array}{cccc} -2 & -1 & 0 & -\rho_{1}\\ -1 & -2 & -\rho_{2} & 0\\ \sqrt{2} & 1 & -2 & 0\\ 1 & \sqrt{2} & 0 & -2 \end{array}\right).$$

It is straightforward to compute that the characteristic polynomial of this matrix is given by

$$\begin{array}{rl} & \det(\text{M}-\lambda \text{I})=(1+\lambda ){\left(2+\lambda \right)}^{2}(3+\lambda )+\left(\lambda^{2}+\left(4-\sqrt{2}\right)\lambda +\left(4-2\sqrt{2}\right)\right)\rho_{1}\\ & \;+\left(\lambda^{2}+\left(4-\sqrt{2}\right)\lambda +\left(4-2\sqrt{2}\right)\right)\rho_{2}-\rho_{1}\rho_{2}. \end{array}$$

The zero eigenvalue curve is given by

$$\begin{array}{rl} & 12+\left(4-2\sqrt{2}\right)\rho_{1}+\left(4-2\sqrt{2}\right)\rho_{2}-\rho_{1}\rho_{2}=0\\ \;\mathrm{and}\; & \;\rho_{1}=\frac{12+\left(4-2\sqrt{2}\right)\rho_{2}}{\rho_{2}-\left(4-2\sqrt{2}\right)}. \end{array}$$

The bifurcation curve is given by the envelope

$$\begin{array}{rl} \rho_{1} & =-(\lambda +2)\left(\lambda +2+\frac{\sqrt{2}}{2}\right)\pm (\lambda +2)\sqrt{2\left(\lambda +\frac{3}{2}\right)\left(\lambda +\frac{5}{2}\right)}\\ \;\mathrm{and}\;\rho_{2} & =-(\lambda +2)\left(\lambda +2+\frac{\sqrt{2}}{2}\right)\mp (\lambda +2)\sqrt{2\left(\lambda +\frac{3}{2}\right)\left(\lambda +\frac{5}{2}\right)} \end{array}$$

together with the singular piece $\rho_{1}=-\frac{1}{2}\cup \rho_{2}=-\frac{1}{2}$, which is associated with the value $\lambda =-2+\sqrt{2}/2$, where the equations defining the envelope fail to have full rank. The envelope and the singular piece of the bifurcation curve meet tangentially at $\left(\rho_{2}=-\frac{1}{2},\rho_{1}=-\frac{3}{2}\right)$ and $\left(\rho_{2}=-\frac{3}{2},\rho_{1}=-\frac{1}{2}\right)$. Because of the symmetry we have *P*_1_=*P*_2_ and thus *P*_1_∧*P*_2_∧*Q*≡0, so the condition for a triple eigenvalue reduces to simultaneous vanishing of *P*_1_∧*D*∧*Q* and *D*∧*P*_2_∧*Q*. Note that since *P*_1_=*P*_2_ these are not independent—*P*_1_∧*D*∧*Q*=−*D*∧*P*_2_∧*Q*. Calculating we find that the triple eigenvalue condition becomes

$$P_{1}\wedge D\wedge Q=-16\lambda^{3}+\left(12\sqrt{2}-96\right)\lambda^{2}+\left(48\sqrt{2}-192\right)\lambda +\left(46\sqrt{2}-128\right)=0.$$

This cubic has three real roots: a double root at $\lambda =-2+\sqrt{2}/2$ and a simple root at $\lambda =-\left(\frac{1}{2}+\sqrt{2}/4\right)\approx -2.35$. The envelope is not defined for $\lambda \in \left(-\frac{5}{2},-\frac{3}{2}\right),$ so the root at $\lambda =-\left(\frac{1}{2}+\sqrt{2}/4\right)$ does not correspond to a real multiple eigenvalue. Thus, the only real eigenvalue of multiplicity higher than two is $\lambda =-2+\sqrt{2}/2$. Since this eigenvalue is associated with the singular piece of the bifurcation curve we can potentially have many points where this is a triple eigenvalue. Along the curve $\rho_{1}=-\frac{1}{2}$ the eigenvalues are

$$\lambda =-2+\frac{\sqrt{2}}{2},-2+\frac{\sqrt{2}}{2},-2-\frac{\sqrt{2}}{2}\pm \frac{\sqrt{2-4\rho_{2}}}{2}.$$

So the only triple eigenvalue is at $\rho_{2}=-\frac{3}{2}$, the point of intersection with the envelope curve. A similar calculation holds along $\rho_{2}=-\frac{1}{2}$.

The Hopf curve is given parametrically by

2.18 $$\rho_{1}=\left(4+\sqrt{2}\right)\frac{(4\omega^{2}-14)\pm \sqrt{30\left(18-8\sqrt{2}+\left(1-2\sqrt{2}\right)\omega^{2}+\omega^{4}\right)}}{14}$$

and

**Figure 2.**
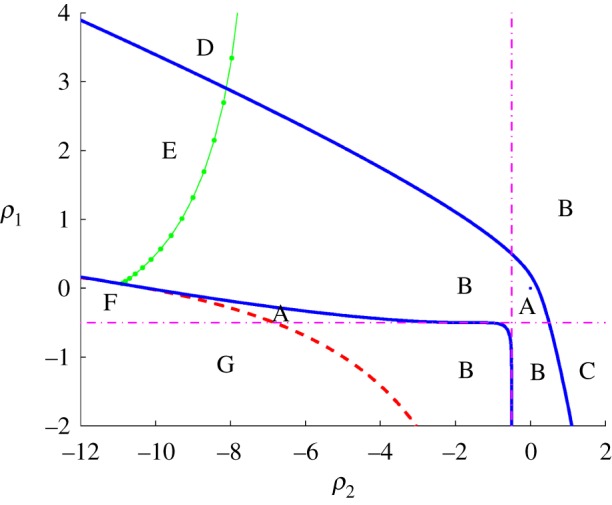
The stability diagram in the (*ρ*_2_,*ρ*_1_) plane for the model introduced in example 2.7. The bifurcation (blue), Hopf (green dotted) and zero eigenvalue (red dashed) curves are shown. The bifurcation curve also has a singular piece (magenta dot-dashed), for $\lambda =-2+\sqrt{2}/2$, where the equations defining the envelope curve fail to have full rank.

2.19 $$\rho_{2}=\left(4+\sqrt{2}\right)\frac{(4\omega^{2}-14)\mp \sqrt{30\left(18-8\sqrt{2}+\left(1-2\sqrt{2}\right)\omega^{2}+\omega^{4}\right)}}{14},$$

where, as always, the signs are *not* independent. Note that the argument of the square root is strictly positive, so there exist purely imaginary eigenvalues corresponding to oscillations of any desired frequency. The Hopf curve, the envelope and the zero eigenvalue line all meet at the points $(\rho_{1}=(4+\sqrt{2})(-1\pm \sqrt{30(18-8\sqrt{2})}/14)=4+\sqrt{2}\pm \sqrt{30},\rho_{2}=(4+\sqrt{2})(-1\mp \sqrt{30(18-8\sqrt{2})}/14)=-(4+\sqrt{2})\mp \sqrt{30}$. The most interesting region of the stability diagram is depicted in figure 2. The zero eigenvalue curve is depicted in dashed red, the envelope in blue (including a dot at the origin), the singular piece of the bifurcation curve in dot-dashed magenta and the Hopf curve in solid dotted green.

From this information, it is easy to derive the stability diagram. At the origin, the eigenvalues are λ=−3,λ=−2,λ=−2,λ=−1. As there is a degenerate eigenvalue one needs to do a local perturbation analysis near λ=−2,*ρ*_1_=0,*ρ*_2_=0 to determine whether in the neighbourhood of this point one has a real pair of eigenvalues or a complex conjugate pair. Letting λ=−2+*δ* shows that near this point one has

$$\det(\text{M}-\lambda \text{I})=-\delta^{2}-\sqrt{2}\delta \rho_{1}-\delta \sqrt{2}\rho_{2}-\rho_{1}\rho_{2}+O(3),$$

where *O*(3) denotes terms of order three or higher in *δ*,*ρ*_*i*_. The discriminant of the above is $(\sqrt{2}\rho_{1}+\sqrt{2}\rho_{2}{)}^{2}-4\rho_{1}\rho_{2}=2\rho_{1}^{2}+2\rho_{2}^{2}>0,$ indicating that in a neighbourhood of the origin the double eigenvalue splits into a real (distinct) pair of eigenvalues. Thus in the region containing the origin and bounded by the singular pieces of the bifurcation curve and the upper branch of the envelope (labelled **A**) there are four real eigenvalues in the left half-plane (LHP). As *ρ*_2_ is decreased so as to cross the line $\rho_{2}=-\frac{1}{2}$ the first bifurcation occurs. Since this line corresponds to eigenvalue $\lambda =-2+\sqrt{2}$ and $-2<-2+\sqrt{2}/2<-1$ the bifurcation consists of the two dominant real eigenvalues bifurcating to a complex conjugate pair. Thus in region **B** we have two real eigenvalues and two complex eigenvalues, all in the left half-plane. As one leaves region **B** across the Hopf curve into the region labelled **E** the complex conjugate pair moves into the right half-plane (RHP), giving two complex eigenvalues in the right half-plane and two real eigenvalues in the left half-plane. Proceeding in this manner the stability diagram can be labelled as follows:

— Region A: four real eigenvalues in the left half-plane.
— Region B: two real and two complex eigenvalues in the left half-plane.
— Region C: four complex eigenvalues in the left half-plane.
— Region D: two complex eigenvalues in right half-plane, two complex eigenvalues in the left half-plane.
— Region E: two complex eigenvalues in the right half-plane, two real eigenvalues in the left half-plane.
— Region F: one real eigenvalue in the right half-plane, three real eigenvalues in the left half-plane.
— Region G: one real eigenvalue in the right half-plane, one real and two complex eigenvalues in the left half-plane.

Additionally, there is a narrow region between the regions labelled **E** and **F** (to the left of $\rho_{2}=-\left(4+\sqrt{2}\right)-\sqrt{30}\approx -10.9$ above the zero eigenvalue curve and below the envelope curve) where there are two real eigenvalues in the left half-plane and two real eigenvalues in the right half-plane. This region is not labelled since it is not visible on this scale.

One feature which we have not labelled are points where a real eigenvalue and a complex conjugate pair all have the same real part, corresponding to points where a real eigenvalue and a complex conjugate pair exchange dominance. Although it is easy to write down an implicit equation satisfied by these curves, it is generally not possible to find an explicit formula as for the Hopf curve, envelope, etc.

As the characteristic polynomial of this problem is of order four, it would, in principle, be possible to extract the above information directly from the solution formula for the quartic. In practice, it would be exceedingly difficult to recover such detailed information. In the next section, we consider a model that arises from a differential equation of order *eight*; in this situation, it is no longer possible even in principle to state a direct formula for the roots.

In this section, we identified—and gave a recipe for computing—a set of geometric quantities associated with the spectrum of a rank-two perturbation of a well-known operator. Because some of these quantities (specifically the bifurcation curve, the λ=0 eigenvalue curve, and the Hopf curve) form curves which together partition the (*ρ*_1_,*ρ*_2_) plane into open sets with qualitatively similar behaviour, this information allows us to analyse the perturbed operator in the entire plane. We now apply this procedure to three specific problems which can be written in the form of equation (1.1): a model of a coupled brainstem–cerebellum neuronal network called the *oculomotor integrator* (§3); a continuum version of that model, in which the (relatively numerous) brainstem neurons are replaced by a neural ‘line’ (§4); and a stability problem that arises in a non-local reaction–diffusion equation [2] (§5).

## 3. Example: a model for the oculomotor integrator

The *oculomotor integrator* is a neural network that is essential for eye movement control. This network holds your gaze steady despite transient body motions, by using cues it receives from oculomotor subsystems such as the vestibular system (which processes sensory input from the semi-circular canals of the inner ear) [13,14]. Here, we present a model for the oculomotor integrator which can either simulate normal integrator function, or a common eye movement disorder—congenital or *infantile nystagmus* (IN)—with a few small changes. We are able to make this determination by analysis of the envelope, constant eigenvalue curves and Hopf curve as described in §2. Some of this analysis was presented in [15], which focused on using the model to simulate various eye movements associated with IN; here, we focus on analysing the full-phase space generated by the low-rank perturbations.

We first summarize how the integrator works. The neurons that compose the integrator are located mainly in a brainstem region known as the *vestibular nucleus*. These neurons must integrate *velocity* signals into a desired *position*; thus, they perform the operation of integration with respect to time (or temporal integration). Single neurons produce a small amount of temporal integration, in that a firing rate increase due to a transient input will decay with a time constant of about 5 ms. The observed integrator time constant is closer to 20 s, so the integrator network must lengthen the single-neuron time constant by about 4000 times [14]; for a linear system, this corresponds to having an eigenvalue near zero [16]. Furthermore, the integrator should have the right *gain* in response to velocity signals; the ratio of its response to the input should be appropriate. It should also be *plastic*; i.e. it should be able to adjust, if injury or some other change occurs (for example, you adjust your oculomotor integrator gain when you get a new pair of glasses) [17–20].

In order for the oculomotor integrator to function, the vestibular nucleus must be connected to a second major brain region, the *cerebellum* [21–23]. These connections are essential both for normal operation and for plasticity [24–27]. Neuroanatomical research has shown that these connections are asymmetric in an important way: while the connections from the vestibular nucleus to the cerebellum are numerous and excitatory, the feedback connections from the cerebellum are sparse and are inhibitory [28–33]. Anastasio & Gad [1] showed previously that this asymmetry permits the cerebellum to sensitively control both the time constant and the gain of the oculomotor integrator, despite (or perhaps because) the projections from the cerebellum back to the vestibular nuclei are so sparse. *These sparse cerebellar- to-vestibular connections, which can plastically change their strength, are precisely the low-rank perturbations we analyse in detail here.*

Anastasio & Gad [1] proposed a linear differential equation model that combined a vestibular network with sparse, asymmetric feedback connections from cerebellar *Purkinje cells*, i.e. the evolution of the system is given by:

3.1 $$\frac{dv}{dt}=\tilde{\text{M}}v+s(t)b\;v(0)=\text{0},$$

where ***v*** represents the response of the system (vestibular neurons and Purkinje cells together), $\tilde{\text{M}}$ represents a matrix of connections, *s*(*t*) is a scalar input (velocity) signal to the integrator and ***b*** a fixed vector representing the pattern in which the vestibular neurons receive the input signal.

The connection matrix $\tilde{\text{M}}$ proposed by [1] was

3.2 $$\tilde{\text{M}}=\alpha \left(\begin{array}{ccc} \text{T} & -\rho_{1}u_{1} & -\rho_{2}u_{2}\\ w_{1}^{t} & -1 & 0\\ w_{2}^{t} & 0 & -1 \end{array}\right).$$

The vestibular neuron-to-Purkinje cell coupling vectors are given by the ***w***_*i*_; the Purkinje cell-to-vestibular neuron coupling vectors are given by (***u***_1_)_*j*_=*δ*_*j*,*k*_1__,(***u***_2_)_*j*_=*δ*_*j*,*k*_2__, where *δ*_*j*,*k*_ is the Kronecker delta.

We state the remaining details for completeness; for a full explanation of the biophysical motivation, see [1,15]. The matrix **T** of effective connections between vestibular neurons is given by

3.3 $$\text{T}=\left(\begin{array}{cccccc} -1+\beta & \beta & 0 & 0 & \cdots & 0\\ \beta & -1+\beta & \beta & 0 & \cdots & 0\\ \vdots & \vdots & \vdots & \vdots & \ddots & \vdots \\ 0 & 0 & 0 & 0 & \beta & -1+\beta \end{array}\right).$$

The parameter *α* is set to 200 *s*^−1^ (corresponding to a typical 5 *ms* membrane time constant); the parameter *β* is fixed so that the vestibular sub-network has a time constant of 0.2 *s* in the absence of cerebellar interaction (*ρ*_1_=*ρ*_2_=0). The largest eigenvalue of **T** is given by $\lambda_{1}/\alpha =-1+\beta (1+2\cos\;(\pi /(N+1)))$, where *N* is the number of vestibular cells (on each side of the network); therefore, we choose $\beta =(\lambda_{1}/\alpha +1)/(1+2\cos\;(\pi /(N+1)))$, where λ_1_=5 *s*^−1^.

As we have noted, we will treat the Purkinje-to-vestibular feedback connections as our low-rank perturbations; to relate this model to equation (1.1), take

3.4 $$\text{M}=\alpha \left(\begin{array}{ccc} \text{T} & \text{0} & \text{0}\\ w_{1}^{t} & -1 & 0\\ w_{2}^{t} & 0 & -1 \end{array}\right);\;f_{i}=-\rho_{i}\left[\begin{array}{c} u_{i}\\ 0\\ 0 \end{array}\right],\;i=1,2;\;{\left(g_{i}\right)}_{j}=\delta_{j,N+i},\;i=1,2,$$

where *N* is the number of vestibular neurons (therefore **M** is (*N*+2)×(*N*+2), and ***f***_*i*_, ***g***_*i*_ are in $R^{N+2}$). We will assume that the output of the integrator is a linear readout of the vestibular neuron responses, which for simplicity we take to be equal to ***b***: i.e. 〈***b***,***v***(*t*)〉. Defining the eigenvectors ***e***_*i*_ and adjoint eigenvectors ***f***_*i*_, respectively, by

$$\begin{array}{r} \tilde{\text{M}}e_{i}=\lambda_{i}e_{i}\;\mathrm{and}\;{\tilde{\text{M}}}^{t}f=\lambda_{i}f_{i}, \end{array}$$

the linear readout at time *t* (assuming $\tilde{\text{M}}$ diagonalizable) is given by

$$\langle b,v(t)\rangle =\sum_{i}\frac{\left\langle b,e_{i}\rangle \langle \;f_{i},b\right\rangle }{\left\langle \;f_{i},e_{i}\right\rangle }\int_{0}^{t}e^{\lambda_{i}(t-t^{\prime })}s(t^{\prime })\;dt^{\prime }.$$

When there is a separation of time scales (Re(λ_2_)≪Re(λ_1_)), the response is largely due to the dominant eigenvalue; we define the *gain*, *γ*, to be the ratio of this response, to the magnitude of the filtered input:

3.5 $$\begin{array}{r} \langle b,v(t)\rangle \approx \frac{\left\langle b,e_{1}\rangle \langle \;f_{1},b\right\rangle }{\left\langle \;f_{1},e_{1}\right\rangle }\int_{0}^{t}e^{\lambda_{1}(t-t^{\prime })}s(t^{\prime })\;dt^{\prime } \end{array}$$

3.6 $$\begin{array}{r} =\frac{\left\langle b,e_{1}\rangle \langle \;f_{1},b\right\rangle }{\langle \;f_{1},e_{1}\rangle ∥b{∥}^{2}}\times \left[∥b{∥}^{2}\int_{0}^{t}e^{\lambda_{1}(t-t^{\prime })}s(t^{\prime })\;dt^{\prime }\right] \end{array}$$

3.7 $$\begin{array}{r} =\gamma \left[∥b{∥}^{2}\int_{0}^{t}e^{\lambda_{1}(t-t^{\prime })}s(t^{\prime })\;dt^{\prime }\right]. \end{array}$$

Thus, *γ* captures how the circuit amplifies—or suppresses—the incoming signal.

Setting this ratio correctly allows the organism to respond appropriately to its environment. However, injury or normal growth can alter the sensory systems that supply inputs to the integrator, and thus create a mismatch between input and desired output. To counteract this, the system must change this gain to compensate. We will allow our network to change, by adjusting the Purkinje-to-vestibular weights *ρ*_1_ and *ρ*_2_; this is biologically plausible, since one of the major functions of the cerebellum is to regulate motor plasticity and learning.

The asymmetry of the matrix $\tilde{\text{M}}$ is crucial, to allow gain to be adjusted freely; for a symmetric (more generally *normal*) matrix, −1≤*γ*≤1. When can we get big changes in gain, with relatively small changes in *ρ*_1_ and *ρ*_2_? It would be ideal for the denominator of *γ* to be near zero (assuming both ***f***_1_, ***e***_1_ are normalized to unit length): 〈***f***_1_,***e***_1_〉≪1. However, this corresponds to near-orthogonality of the right and left eigenvectors, which occurs near a double eigenvalue (perfect orthogonality, 〈***f***_1_,***e***_1_〉=0, can occur only when there is a degenerate double eigenvalue).

We now show an example of an integrator that can perform normal integration with arbitrary adjustment of gain: let **T** be 6×6 and choose

3.8 $$\begin{array}{rl} u_{1} & =e_{1},\;u_{2}=e_{3},\\ w_{1}^{t} & =[-1\;1\;-1\;0\;-1\;0]\\ \;\text{and}\;w_{2}^{t} & =[1\;-1\;1\;1\;0\;0], \end{array}\}$$

where ***e***_*j*_ is the *j*th identity vector (in this case $e_{j}\in R^{6}$).

With this choice, $\tilde{\text{M}}$ is the reduced matrix of a model with six vestibular neurons and two Purkinje cells on each side of the bilaterally symmetric network. The only parameters that may vary are *ρ*_1_ and *ρ*_2_, the strengths of the Purkinje-to-vestibular connections. In order to fix a certain time constant, *ρ*_2_ and *ρ*_1_ should be constrained to lie on the appropriate constant eigenvalue curve. The biologically appropriate time constant is in the neighbourhood of 20 *s*, so the appropriate eigenvalue is $\lambda =-\frac{1}{20}.$ From the results of §2, we know that this holds along the curve

$$Q\left(-\frac{1}{20}\right)\rho_{1}\rho_{2}+P_{2}\left(-\frac{1}{20}\right)\rho_{2}+P_{1}\left(-\frac{1}{20}\right)\rho_{1}+D\left(-\frac{1}{20}\right)=0,$$

or (approximately)

3.9 $$\rho_{1}=\frac{0.137+2.536\rho_{2}}{1+0.371\rho_{2}}.$$

This constant eigenvalue curve is tangent to the envelope at the simultaneous solution of

$$\begin{array}{rl} 0.137-\rho_{1}+2.536\rho_{2}-0.371\rho_{1}\rho_{2} & =0\\ \;\mathrm{and}\;0.577-\rho_{1}+2.405\rho_{2}-0.473\rho_{1}\rho_{2} & =0. \end{array}$$

(Note: we have divided each equation through by a constant.) The biologically important root of the above pair of equations is the one in the first quadrant, (*ρ*_2_,*ρ*_1_)=(1.22,2.23): negative values of *ρ* would correspond to an *excitatory* Purkinje-to-vestibular connection, which is not known to occur.

The basic picture of integrator operation is as follows: let us suppose that *ρ*_2_ is allowed to vary but that *ρ*_1_ is given by equation (3.9), so that $\lambda =-\frac{1}{20}$ is always an eigenvalue. As *ρ*_2_ is increased from zero the dominant eigenvalue is fixed at $\lambda =-\frac{1}{20}$ and the (in this case real) subdominant eigenvalue increases. Because we are nearing the envelope curve (and thus a degenerate double eigenvalue $\lambda_{2}=\lambda_{1}=-\frac{1}{20}$), we expect the gain to increase. At *ρ*_2_=1.22, where the constant eigenvalue curve is tangent to the envelope, there is a collision of eigenvalues and the dominant eigenvalue is degenerate. As *ρ*_2_ is further increased the formerly subdominant eigenvalue is now dominant—it is real and larger than $-\frac{1}{20}$. Furthermore, we can compute the gain associated with the dominant mode along the $\lambda =-\frac{1}{20}$ curve and find

3.10 $$\gamma =\frac{\left\langle \;f_{1},b\rangle \langle b,e_{1}\right\rangle }{∥b{∥}^{2}\langle \;f_{1},\;e_{1}\rangle }\approx \frac{0.05(\rho_{2}+1.43)(\rho_{2}+1.86)}{\left(1.22-\rho_{2})(1.65+\rho_{2}\right)}.$$

Note that, as expected, the denominator of the gain diverges at *ρ*_2_=1.22, where the constant eigenvalue curve is tangent to the envelope and the $\lambda =-\frac{1}{20}$ eigenvalue is degenerate.

**Figure 3.**
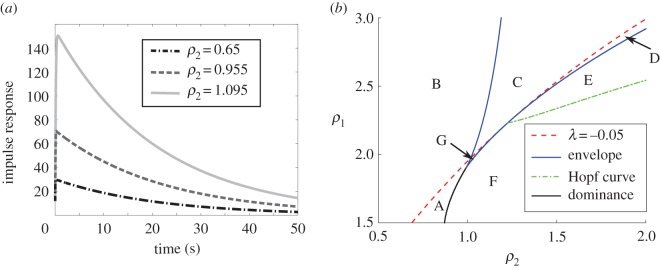
(*a*) Impulse response of network as network attempts to increase gain while maintaining λ_*dominant*_. (*b*) Phase space of network showing normal operation. Letters indicate the relative positions of the *three* most dominant eigenvalues. Region A: 1 real (dominant), 2 complex in the left half-plane (LHP). Region B: 1 real (dominant) in the right half-plane (RHP), 2 complex in the LHP. Region C: 1 real (dominant) in the RHP, 2 real in the LHP. Region D: 2 real (dominant) in the RHP, 1 real in the LHP. Region E: 2 complex (dominant) RHP, 1 real LHP. Region F: 2 complex (dominant) LHP, 1 real LHP. Region G: 3 real LHP. We note that λ_*dominant*_ is real unstable in B,C,D; complex unstable in E; complex stable in F; real stable in A,G.

In figure 3*a*, we show the response of the network to an impulsive forcing of the form *f*(*t*)=*δ*(*t*)(1,1,1,1,1,1,0,0)^*t*^ as *ρ*_2_, *ρ*_1_ are varied to increase gain. (Note that the impulsive forcing is equivalent to free decay with a corresponding initial condition.) Three responses are shown, with *ρ*_2_ set to 0.65, 0.955 and 1.095, respectively. For each value of *ρ*_2_, *ρ*_1_ is set so that the network lies on the constant time constant curve *τ*=20 *s*. The gains predicted based on a single dominant mode (*γ* in equation (3.10)) are 2.52,5.92 and 12.88. The corresponding measurements from the impulse response (given by dividing the maximum response by ∥***b***∥^2^) yielded 2.54, 5.87 and 12.53, respectively; so we see excellent agreement.

Figure 3*b* displays the interaction of the two dominant eigenvalues of the network in the vicinity of the current operating region. In order to increase gain, the network must climb up the λ=−0.05 curve in the vicinity of the double eigenvalue point. At the intersection of this curve with the envelope, the integrating eigenvalue exchanges dominance with another real eigenvalue, producing an unstable integrator. Note that the larger two gain cases straddle the point where the $\lambda =-\frac{1}{20}\;s^{-1}$ curve crosses the envelope, indicating an eigenvalue bifurcation in a subdominant mode. In this case, it is the mode(s) with the next largest real part. At about *ρ*_2_≈1.02, the subdominant complex conjugate pair collides at the real axis, and for *ρ*_2_ above this value the first three most dominant eigenvalues are all real. As *ρ*_2_ increases the subdominant eigenvalue increases until *ρ*_2_≈1.22 where there is an eigenvalue collision and exchange of dominance.

Figure 3*b* also uses letter labels to show the character of the dominant eigenvalue. Normal operation requires the network to remain in Regions A or G. If the network is in error, it may wander into a region where the two eigenvalues are complex (F), or into the region where one or both eigenvalues are in the right half-plane (B,C, D or E). In neither case is normal operation possible.

*Infantile nystagmus* (IN) is a hereditary disorder characterized by involuntary, periodic eye movements. These movements (or waveforms) can be broadly classified into two forms, *jerk* and *pendular*. In jerk waveforms, the eye moves outward from the central position with increasing speed until interrupted by a sudden saccade; a pendular waveform resembles a sinusoidal oscillation. The presence of a jerk waveform suggests an unstable eigenvalue (λ>0); as noted in many integrator models, the need to maintain an eigenvalue very near zero implies that an unstable eigenvalue is a natural consequence of imprecision.

In our model, the ability to modulate gain is enhanced near the bifurcation curve; this suggests that the network is *also* near a point where the dominant eigenvalue is complex, which would generate the sinusoidal oscillations characteristic of pendular nystagmus. For example, consider the network specified in equation (3.11). This is very similar to equation (3.8); the only change is that the vestibular-to-Purkinje input from a handful of neurons has been altered. Here, as the cerebellum attempts to increase gain by adjusting *ρ*_1_ and *ρ*_2_, it enters an oscillating regime.

3.11 $$\begin{array}{rl} u_{1} & =e_{1},\;u_{2}=e_{3},\\ w_{1}^{t} & =[-1\;1\;0\;0\;-1\;0]\\ \;\text{and}\;w_{2}^{t} & =[1\;-1\;0\;0\;1\;0] \end{array}\}$$

**Figure 4.**
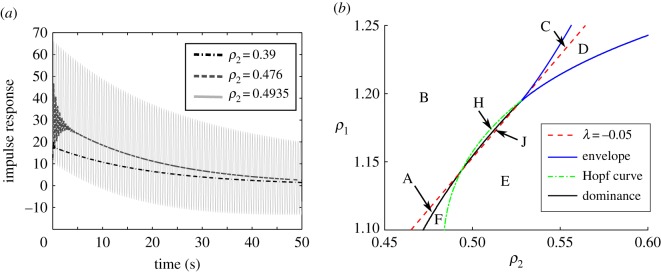
(*a*) Impulse response of network as network attempts to increase gain while maintaining λ_*dominant*_. (*b*) Phase space of network showing pendular nystagmus. Letters indicate the relative positions of the *three* most dominant eigenvalues; labels are as in figure 4, with the addition of new regions ‘H’ and ‘J’. Region A: 1 real (dominant), 2 complex in the LHP. Region B: 1 real (dominant) in the RHP, 2 complex in the LHP. Region C: 1 real (dominant) in the RHP, 2 real in the LHP. Region D: 2 real (dominant) in the RHP, 1 real in the LHP. Region E: 2 complex (dominant) RHP, 1 real LHP. Region F: 2 complex (dominant) LHP, 1 real LHP. Region H: 1 real (dominant) and 2 complex in the RHP. Region J: 2 complex (dominant) and 1 real in the RHP. (There is no Region G: 3 real LHP here.) We note that λ_*dominant*_ is real unstable in B,C,D,H; complex unstable in E,J; complex stable in F; real stable in A.

We see the impulse response of the network in figure 4*a*. As in figure 3*a*, *ρ*_2_ and *ρ*_1_ are varied so as to remain along the $\lambda =-\frac{1}{20}\;s^{-1}$ curve. As *ρ*_2_ (and the gain) increase, the network enters a regime where an oscillation is superimposed on normal integration. Figure 4*b* illustrates why this behaviour occurs. As we follow the $\lambda =-\frac{1}{20}\;s^{-1}$ curve from left to right through Region A, gain will increase. However, the eigenvalue curve has an intersection with the Hopf curve far to the left of its intersection with the envelope. At this point, the integrating eigenvalue exchanges dominance with a pair of imaginary eigenvalues. Beyond this point, the response of the network contains both the normal integrating mode and a superimposed oscillation (Region F).

## 4. Example: a continuum model of the oculomotor integrator

We next consider a continuum model that can be derived from the model of Anastasio and Gad by replacing the (relatively numerous) vestibular neurons with a continuum vestibular ‘line’ denoted by *ψ*(*x*), while the relatively few Purkinje cells remain discrete.

Suppose the network has *N* vestibular cells on each side of the integrator, arranged in a row of length *L*. Recall that the vestibular interaction matrix **T** has nearest-neighbour structure; each row of **T** takes the form *βv*_*j*−1_+(−1+*β*)*v*_*j*_+*βv*_*j*+1_ (equation (3.3)). This will converge, in the continuum limit (N→∞), to a second derivative, because:

$${\left(\frac{L}{N}\right)}^{2}\psi_{xx}=\psi_{i+1}+\psi_{i-1}-2\psi_{i}+o\left({\left(\frac{L}{N}\right)}^{3}\right).$$

The sum over vestibular cells in the equation for the Purkinje cells (two final rows of equation (3.2)) can similarly be replaced by an integral:

$$\sum_{j}{\left(w_{1}^{t}\right)}_{j}v_{j}\to \frac{1}{L/N}\int_{0}^{L}\psi (x)\phi_{1}(x)\;dx,$$

where *ψ*(*jΔx*)=*v*_*j*_ and $\phi_{1}(j\Delta x)={\left(w_{1}^{t}\right)}_{j}$.

This leads to the following integro-differential eigenvalue problem on $L_{2}[0,L]\times R^{2}$

4.1 $$\beta {\left(\Delta x\right)}^{2}\psi_{xx}+(-1+3\beta )\psi -\rho_{1}P_{1}\delta (x-x_{1})-\rho_{2}P_{2}\delta (x-x_{2})=\lambda \psi ,\;\psi (0)=0=\psi (L)$$

and

4.2 $$-P_{i}+\frac{1}{\Delta x}\int_{0}^{L}\psi (x)\phi_{i}(x)\;dx=\lambda P_{i},\;i=1,2$$

where we have replaced *L*/*N* with *Δx*.^2^ The delta function coupling reflects the sparseness of the Purkinje-to-vestibular connections, with *x*_1,2_ denoting the points on the vestibular line innervated by the Purkinje cells, and the functions *ϕ*_1,2_(*x*) represent the density of vestibular to Purkinje connections.

Eliminating *P*_1_, *P*_2_ from equation (4.1) algebraically, we arrive at the single equation

4.3 $$\begin{array}{rl} & \psi_{xx}+\frac{-1+3\beta }{\beta {\left(\Delta x\right)}^{2}}\psi -\frac{\rho_{1}\langle \psi ,\phi_{1}\rangle }{\beta {\left(\Delta x\right)}^{3}(\lambda +1)}\delta (x-x_{1})-\frac{\rho_{2}\langle \psi ,\phi_{2}\rangle }{\beta {\left(\Delta x\right)}^{3}(\lambda +1)}\delta (x-x_{2})\\ & \;=\frac{\lambda }{\beta {\left(\Delta x\right)}^{2}}\psi ,\;\psi (0)=0=\psi (L). \end{array}$$

This maps to our original problem, equation (1.1), as follows:

4.4 $$\text{M}\psi =(\beta {\left(\Delta x\right)}^{2}\psi_{xx}+(-1+3\beta ))\psi ;\;f_{i}=\frac{\delta (x-x_{i})}{\Delta x(\lambda +1)},\;i=1,2;\;g_{i}=\phi_{i},\;i=1,2.$$

This model can be solved in much the same way as the discrete model analysed in §3. To illustrate we take *L*=1 and the vestibular-to-Purkinje connections to be *ϕ*_1_(*x*)=*ϕ*_2_(*x*)=1. Note that when *ρ*_1_=0,*ρ*_2_=0, the vestibular neurons decouple from the Purkinje cells, and the eigenvectors *ψ* are given by trigonometric functions with the appropriate boundary conditions:

4.5 $$\psi_{n}(x)=\sin\;(n\pi x),$$

after which *P*_1_, *P*_2_ are obtained by using equations (4.2):

4.6 $$P_{1}=P_{2}=\frac{N^{2}(1-\cos\;(n\pi ))}{n\pi (3\beta -\beta {\left(n\pi /N\right)}^{2})}.$$

The corresponding eigenvalues are given by λ_*n*_=−1+3*β*−*β*(*n*^2^*π*^2^/*N*^2^), together with λ=−1, an eigenvalue of multiplicity two corresponding to the Purkinje cells. Note that the even modes (*n*=2*k*) do not actually excite a Purkinje cell response (i.e. *P*_1_=*P*_2_=0), and thus the even modes are eigenfunctions of this problem for all values of *ρ*_1_,*ρ*_2_: these modes do not change under perturbation by the Purkinje cells.

We now apply the techniques developed earlier in the paper to find the lines of constant eigenvalue. We will not reproduce the entire calculation, but merely note a few salient points. The first step is to act on equation (4.1) with the appropriate resolvent (inverse) operator (here, *R*_λ_=(*β*(*Δx*)^2^∂_*xx*_+3*β*−(1+λ))^−1^). The terms *R*_λ_*δ*(*x*−*x*_*i*_) are simply Green’s function for the operator *β*(*Δx*)^2^∂_*xx*_+3*β*−(1+λ) acting on *L*_2_[0,*L*] with Dirichlet boundary conditions, which can be easily calculated—see (for example) the text of Keener [34]; we also supply some details in appendix A.

The main conclusion is that the perturbed problem will have piecewise trigonometric eigenfunctions of the form sin (ωx), etc. (with appropriate continuity conditions; see equation (A.16)), where

4.7 $$\omega^{2}=\frac{-\lambda -1+3\beta }{\beta {\left(\Delta x\right)}^{2}}\;\Rightarrow \;\lambda =-1+3\beta -\beta {\left(\Delta x\right)}^{2}\omega^{2}.$$

We now fix the remaining constants in the model: $x_{1}=\frac{1}{3}$ and $x_{2}=\frac{1}{2}$, and perform the calculation thus described. We find that the resulting envelope curves have asymptotes when *ω* is a multiple of 4*π* and 6*π*; this is a consequence of the location of the Purkinje cell innervation (i.e. *x*_1_ and *x*_2_).

One issue that becomes apparent, is that as the network becomes large we are unable to find an integrating eigenvalue using equation (4.7) in this way; observe that λ is bounded above by $\lambda =-1+3\beta -\beta {\left(\Delta x\right)}^{2}\omega^{2}\leq -1+3\beta \approx -\frac{1}{40}+\frac{39}{120}{\left(\pi /(N+1)\right)}^{2}$, which $\to -\frac{1}{40}$ as N→∞. However, the desired eigenvalue for a 20 s time constant (recall that we have scaled out the 5 ms single neuron time constant, *α*=200) is λ=−0.05/200, which is much closer to zero than $-\frac{1}{40}$; as *N* increases, the integrating eigenvalue will become out of reach. One way to view this observation is that as the ratio of vestibular to cerebellar (Purkinje) cells increases, sparse cerebellar innervation becomes less able to alter the time constant of the vestibular network.

The alternative, is to consider piecewise exponential eigenfunctions; i.e.

4.8 $$\psi (x)=\left\{ \begin{array}{ll} A\;\sinh\;\omega x, & x<x_{1}\\ B\;\sinh\;\omega x+C\;\cosh\;\omega x, & x_{1}<x<x_{2}\\ D\;\sinh\;(\omega (L-x)), & x>x_{2}, \end{array}\right.$$

where now

4.9 $$\omega^{2}=\frac{\lambda +1-3\beta }{\beta {\left(\Delta x\right)}^{2}}\Rightarrow \lambda =-1+3\beta +\beta {\left(\Delta x\right)}^{2}\omega^{2}.$$

**Figure 5.**
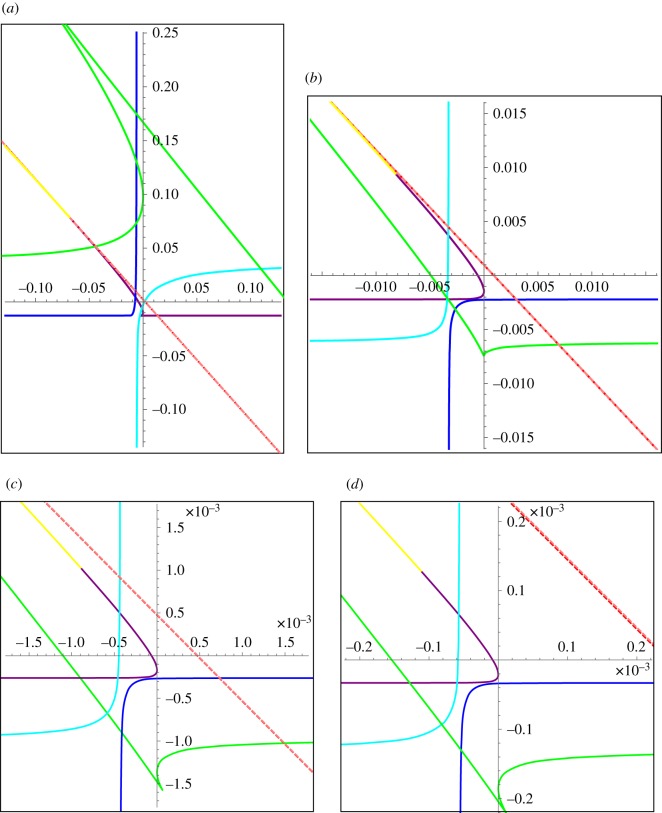
Phase planes for the continuum oculomotor integrator model (equations (4.1) and (4.2)), for several values of *N*. Four pieces of the λ<0 bifurcation curve are shown: (0,4*π*) (purple), (4*π*,6*π*) (blue), (6*π*,8*π*) (cyan), (8*π*,12*π*) (green); λ>0 curve (yellow). Constant eigenvalue curves λ=−0.05/200 (red dashed) and λ=0 (pink dashed) are visually indistinguishable. (*a*) *N*=12, (*b*) *N*=24, (*c*) *N*=50 and (*d*) *N*=100.

Although such functions cannot match the boundary conditions of the *unperturbed* problem, the discontinuities in the perturbed problem bring them back into consideration. We can plot the corresponding bifurcation curve; it begins near (*ρ*_2_,*ρ*_1_)≈(−0.09,0.09) (for *N*=12) and increases without bound into the second quadrant. In figure 5, we plot the phase plane for several values of *N*; *N*=12, 24, 50 and 100. In each panel, we show several pieces of the bifurcation curve in different colours: (0,4*π*) (purple), (4*π*,6*π*) (blue), (6*π*,8*π*) (cyan), (8*π*,12*π*) (green), and the curve for exponential eigenfunctions (yellow). We note that the structure is stable, for increasing *N*; however, the corresponding values of λ are ‘compressed’ (see equation (4.7)). This is to be expected; in the finite-dimensional problem, the eigenvalues cluster together as *N* increases; therefore, we expect increasing density of constant eigenvalue curves as *N* increases.

We now return to the question that originally motivated our analysis in §2; can this network behave as an integrator; i.e. can it achieve the correct time constant and gain, by adjusting its Purkinje-to-vestibular weights *ρ*_1_, *ρ*_2_?

Recall that we characterized the performance of an integrator by two quantities; the dominant eigenvalue λ_1_ and the gain *γ*. In §3, we established that in order to achieve high gain, the parameters *ρ*_1_,*ρ*_2_ must be set near the bifurcation point for the corresponding eigenvalue λ_1_; this is where λ_1_ has multiplicity two, and therefore (if degenerate) where the angle between left and right eigenvectors is near zero. Finally, recall that connections from the cerebellum are always *inhibitory*; by the sign convention in equation (4.3), this means that both *ρ*_1_,*ρ*_2_ must be positive. *Therefore, a necessary condition for the continuum model to act as an integrator, is for the bifurcation point corresponding to λ_1_ to lie in the first quadrant.*

This clearly does not occur in figure 5, where in every panel the yellow curve lies entirely in the second quadrant *ρ*_2_<0, *ρ*_1_>0. Therefore, this network—where *ϕ*_1_=*ϕ*_2_=1, $x_{1,2}=\left\{\frac{1}{3},\frac{1}{2}\right\}$, and plasticity comes from manipulating the strength of the Purkinje-to-vestibular connections *ρ*_1_, *ρ*_2_—cannot regulate both time constant *and* gain.

We now take one further step in generality, by asking how the network behaves as the Purkinje-to-vestibular connection *locations*—*x*_1_ and *x*_2_—change. This would reflect a different source of network plasticity. We will show that for the vestibular-to-Purkinje projection patterns chosen here, the *ρ*_1_,*ρ*_2_ coordinates of the bifurcation point will always be of opposite sign, and therefore will never occur in the first quadrant.

**Lemma 4.1**

*Consider the continuum integrator model defined in equation (4.3) with* *L*=1. *Suppose* *ϕ*_1_=*ϕ*_2_=1; *then for sufficiently large N, the bifurcation point corresponding to the integrating eigenvalue* λ_1_ *will not be found in the first quadrant* (*ρ*_1_,*ρ*_2_>0), *for any* 0<*x*_1_,*x*_2_<1.

**Proof.**

We assume that *N* is large enough that the eigenvalue of interest requires an exponential eigenfunction; i.e. λ_1_>−1+3*β*−*β*(*Δx*)^2^*ω*^2^. We begin by directly computing the required terms in the characteristic polynomial:

4.10 $$\begin{array}{r} D(\omega )=1 \end{array}$$

4.11 $$\begin{array}{r} P_{1}(\omega )=\langle g_{1},R_{\lambda }f_{1}\rangle =\frac{-1+\cosh\;(\omega x_{1})-\sinh\;(\omega x_{1})\tanh\left(\frac{\omega }{2}\right)}{\beta^{2}{\left(\Delta x\right)}^{3}\omega^{2}{\left(3+\omega \Delta x\right)}^{2}} \end{array}$$

4.12 $$\begin{array}{r} \;\text{and}\;P_{2}(\omega )=\langle g_{2},R_{\lambda }f_{2}\rangle =\frac{-1+\cosh\;(\omega x_{2})-\sinh\;(\omega x_{2})\tanh\left(\frac{\omega }{2}\right)}{\beta^{2}{\left(\Delta x\right)}^{3}\omega^{2}{\left(3+\omega \Delta x\right)}^{2}}, \end{array}$$

(refer to equation (2.4) for definitions and appendix A for a more detailed calculation in terms of resolvent operators). Note that

$$P_{1}(\omega )=f(\omega ,x_{1})\;\mathrm{and}\;P_{2}(\omega )=f(\omega ,x_{2}),$$

for the same function *f*(*ω*,*x*).

Then by equations (2.9) and (2.10) the coordinates of the bifurcation point corresponding to any desired *ω*=*ω*_0_ will be:

$$\begin{array}{rl} \rho_{1}(\omega_{0}) & =-\frac{\partial P_{2}}{\partial \omega }(\omega_{0})\times \left(\frac{1}{P_{1}\wedge P_{2}(\omega_{0})}\right)\\ \;\mathrm{and}\;\rho_{2}(\omega_{0}) & =\frac{\partial P_{1}}{\partial \omega }(\omega_{0})\times \left(\frac{1}{P_{1}\wedge P_{2}(\omega_{0})}\right); \end{array}$$

consequently,

$$\frac{\rho_{1}(\omega_{0})}{\rho_{2}(\omega_{0})}=-\frac{\left(\partial f/\partial \omega )(\omega_{0},x_{2}\right)}{\left(\partial f/\partial \omega )(\omega_{0},x_{1}\right)}$$

We will show that (∂*f*/∂*ω*)(*ω*,*x*)>0 for *ω*>0 and 0<*x*<1; therefore, the ratio of *ρ*_1_(*ω*_0_) and *ρ*_2_(*ω*_0_) must be negative.

Differentiating the function *f*(*ω*,*x*) given in equation (4.12), we find that:

4.13 $$\begin{array}{rl} \frac{\partial f}{\partial \omega }(\omega ,x) & =\frac{\left(12+8\Delta x^{2}\omega^{2})(1+\cosh\;(\omega )-\cosh\;(\omega x)-\cosh\;(\omega (1-x))\right)}{\left(1+\cosh\;(\omega )\right)}\\ & \;-2\omega (3+\Delta x^{2}\omega^{2})((1-x)\sinh\;(\omega x)+x\;\sinh\;(\omega (1-x))). \end{array}$$

It may not be obvious what the sign of this function is (the first line is positive and the second negative), but (re-)defining *F*_*ω*_(*x*)≡(∂*f*/∂*ω*)(*ω*,*x*), we will show that *F*_*ω*_(*x*)≥0 for any *ω*>0 and *x*∈[0,1]. We do this by confirming that *F*_*ω*_(*x*) is concave in (0,1), and that *F*_*ω*_(0)=*F*_*ω*_(1)=0. Therefore by the minimum principle for superharmonic functions, *F*_*ω*_(*x*)>0 on the interior *x*∈(0,1).

To confirm concavity, we check that the second derivative in *x* is negative:

4.14 $$\frac{\partial^{2}F_{\omega }}{\partial x^{2}}=\frac{-2\Delta x^{2}\omega [\cosh\;(\omega x)+\cosh\;(\omega (1-x))]-(3+\Delta x^{2}\omega^{2})[x\;\sinh\;(\omega (1-x))+(1-x)\;\sinh\;(\omega x)]}{\beta^{2}\Delta x^{3}{\left(3+\Delta x^{2}\omega^{2}\right)}^{2}(1+\cosh\;(\omega ))}.$$

Checking that *F*_*ω*_(0)=*F*_*ω*_(1)=0 is a simple matter of substituting *x*=0,1 into equation (4.13). ▪

In conclusion, we cannot make this network act as an integrator by adjusting its Purkinje-to-vestibular connections. Instead, we would have to adjust the underlying vestibular-to-Purkinje projection pattern *ϕ*_1_.

## 5. Example: the Rubinstein–Sternberg model

In this section, we give a third, quite different, application for the technique based on low-rank perturbations. Rubinstein & Sternberg [2] introduced a non-local model for phase separation of the form

$$\begin{array}{rl} u_{t} & =\Delta u+f(u)-\frac{1}{|\Omega |}\int_{\Omega }f(u)\;dx,\;(x,t)\in \Omega \times (0,\infty )\\ & \;\text{n}\cdot \nabla u=0,\;x\in \partial \Omega . \end{array}$$

We will compute the stability of a standing front type solution of this model, where the stability operator takes the form of a rank-one perturbation to a standard Sturm–Liouville operator. This model has been analysed by a number of authors, most notably Freitas [3,10,35,36], and the closely related problem of the coarsening rate for the Cahn–Hilliard equation has been analysed in the classic paper of Bates & Fife [37]. The main goal of this example is to illustrate the utility of treating the problem using the rank-one perturbation formula: while the final result appears to be new, several of the intermediary results have analogues in the work of Freitas, and we will point these out where germane.

We begin with the equation

5.1 $$u_{t}=u_{xx}+f(u)-\frac{1}{2L}\int_{-L}^{L}f(u)\;dx,\;u_{x}(\pm L)=0.$$

This equation always admits a constant solution and for sufficiently large widths it admits front type solutions. At $L=n\pi \sqrt{f^{\prime }(0)}/2$, a bifurcation occurs giving rise to a solution containing *n* fronts. In the absence of the non-local term, these front solutions are (for *L* finite) always unstable. The most unstable mode has a non-vanishing mean, so the instability is connected with non-conservation of mass. Rubinstein and Sternberg introduced the non-local term as a Lagrange multiplier to enforce mass conservation and remove this instability mechanism.

It is easy to see that, if *u* represents a stationary solution to equation (5.1) then the linearized evolution equation is given by

5.2 $$v_{t}=v_{xx}+f^{\prime }(u)v-\frac{1}{2L}\int_{-L}^{L}f^{\prime }(u)v\;dx,\;v_{x}(\pm L)=0.$$

Therefore, the associated eigenvalue problem takes the form of a rank-one perturbation of a self-adjoint operator. To explicitly relate equation (5.2) to equation (1.1), we can define

5.3 $$\text{M}v=v_{xx}+f^{\prime }(u)v;\;f_{1}=-\frac{1}{2L};\;g_{1}=f^{\prime }(u);\;f_{2}=g_{2}=0.$$

The problem with a bistable cubic nonlinearity, *f*(*u*)=*u*−*u*^3^,

$$u_{t}=u_{xx}+u-u^{3}-\frac{1}{2L}\int_{-L}^{L}(u-u^{3})\;dx\;u_{x}(\pm L)=0,$$

is the simplest and most natural from a physical perspective, and can be analysed rather explicitly. Assuming *L*>*π*/2, there is a front solution which can be expressed in terms of elliptic functions.^3^ After a simple rescaling (*u*=(1+*k*^2^)^−1^*v*, *x*=(1+*k*^2^)^−1/2^*y* and $t=(\sqrt{2}k/\sqrt{1+k^{2}})s)$, the equation can be written in the form

5.4 $$u_{t}=u_{xx}+(1+k^{2})u-2k^{2}u^{3}-\frac{1}{2K}\int_{-K(k)}^{K(k)}((1+k^{2})u-2k^{2}u^{3})\;dx,\;u_{x}(\pm K(k))=0.$$

Here the quantity *k*∈(0,1) denotes the elliptic modulus and *K*(*k*) denotes the complete elliptic integral of the first kind

$$K(k)=\int_{0}^{1}\frac{dx}{\sqrt{\left(1-x^{2})(1-k^{2}x^{2}\right)}}\in \left(\frac{\pi }{2},\infty \right).$$

The elliptic modulus *k* is determined from *L* by the relation

$$\sqrt{1+k^{2}}K(k)=L.$$

The unperturbed problem now becomes

5.5 $$u_{t}=u_{xx}+(1+k^{2})u-2k^{2}u^{3},\;u_{x}(\pm K(k))=0;$$

a well-known identity for elliptic functions states that a stationary solution to this equation is given by

u(x;k)=sn(x,k)

where sn is the Jacobi elliptic sine function.

Note that as the function sn(*x*,*k*) is odd the non-local term $(1/2K)\int_{-K(k)}^{K(k)}((1+k^{2})u-2k^{2}u^{3})\;dx$ vanishes; thus, this function solves *both* the perturbed and unperturbed problem (i.e. both equations (5.4) and (5.5)). However, the non-local term changes the stability problem; we will find that the stability properties of the two problems are not the same.

Linearizing around the elliptic function solution gives the following non-local evolution equation:

5.6 $$\begin{array}{r} v_{t}=v_{xx}+(1+k^{2})v-6k^{2}{\mathrm{sn}}^{2}(x,k)v-\frac{1}{2K(k)}\int_{-K(k)}^{K(k)}(1+k^{2}-6k^{2}\;{\mathrm{sn}}^{2}(x,k))v\;dx \end{array}$$

5.7 $$\begin{array}{r} =\text{H}v-\frac{1}{2K(k)}\int_{-K(k)}^{K(k)}(1+k^{2}-6k^{2}\;{\mathrm{sn}}^{2}(x,k))v\;dx=:\tilde{\text{H}}v, \end{array}$$

where we have identified $\tilde{\text{H}}$ as a perturbation from a ‘simpler’ operator, **H**. The unperturbed operator **H** is a two-gap Lamé operator, for which the spectral problem can be solved exactly [38,39]. When subject to periodic boundary conditions on [−2*K*(*k*),2*K*(*k*)] the largest five eigenvalues are simple and are given by

5.8 $$\begin{array}{rl} \phi_{0}^{\left(N\right)}(x) & =k^{2}\;{\mathrm{sn}}^{2}(x,k)-\frac{1+k^{2}+a(k)}{3},\;\lambda_{0}^{\left(N\right)}=-(1+k^{2}-2a(k)),\\ \phi_{1}^{\left(D\right)}(x) & =\mathrm{cn}(x,k)\mathrm{dn}(x,k),\;\lambda_{1}^{\left(D\right)}=0,\\ \phi_{1}^{\left(N\right)}(x) & =\mathrm{sn}(x,k)\mathrm{dn}(x,k),\;\lambda_{1}^{\left(N\right)}=-3k^{2},\\ \phi_{2}^{\left(D\right)}(x) & =\mathrm{cn}(x,k)\mathrm{sn}(x,k),\;\lambda_{2}^{\left(D\right)}=-3\\ \;\text{and}\;\phi_{2}^{\left(N\right)}(x) & =k^{2}{\mathrm{sn}}^{2}(x,k)-\frac{1+k^{2}-a(k)}{3},\;\lambda_{2}^{\left(N\right)}=-(1+k^{2}+2a(k)), \end{array}\}$$

with $a(k)=\sqrt{1-k^{2}+k^{4}}$. Here the superscript indicates whether the function satisfies a Neumann or a Dirichlet condition at ±*K*(*k*). Thus, the unperturbed operator subject to Neumann boundary conditions has one positive eigenvalue λ_0_=−(1+*k*^2^−2*a*(*k*)) and the remainder of the eigenvalues on the negative real line.

To summarize, the stability problem (equation (5.7), plus boundary conditions *v*_*x*_(±*K*(*k*))=0) takes the form of a rank-one perturbation of a self-adjoint problem:

$$\tilde{\text{H}}v=\text{H}v+G\langle g,v\rangle ,$$

with *G*=−(1/2*K*(*k*))**1** and *g*=(1+*k*^2^−6*k*^2^ sn^2^(*x*,*k*)). While neither *G* nor *g* is an eigenvector of **H**, both *G* and *g* lie in the span of the zeroth and second eigenfunctions $\phi_{0/2}^{\left(N\right)}(x)=k^{2}\;{\mathrm{sn}}^{2}(x,k)-\frac{1}{3}(1+k^{2}\mp a(k))$. Since the eigenfunctions of a self-adjoint operator are orthogonal this implies that the rank-one piece, *G*〈*g*,*v*〉 vanishes on the span of all the remaining eigenfunctions. This implies that the perturbed operator decomposes as a direct sum of two operators, one that is self-adjoint and negative definite and one that is rank two:

5.9 $$\tilde{\text{H}}=\underset{\mathrm{Rank}\text{-}\mathrm{two}}{\underbrace{\tilde{\text{H}}{|}_{\mathrm{span}(\phi_{0},\phi_{2})}}}⊕\underset{\mathrm{negative}\;\mathrm{definite}}{\underbrace{\tilde{\text{H}}{|}_{\mathrm{span}{\left(\phi_{0},\phi_{2}\right)}^{⊥}}}}=\tilde{\text{H}}{|}_{\mathrm{span}(\phi_{0},\phi_{2})}⊕\text{H}{|}_{\mathrm{span}{\left(\phi_{0},\phi_{2}\right)}^{⊥}}.$$

We emphasize that the perturbation term here is very special, in that *G* and *g* are actually given by linear combinations of just two of the eigenfunctions of the unperturbed operator. In the generic case (i.e. for a different nonlinearity *f*(*u*)), one expects *G* and *g* to have non-trivial projections onto all eigenfunctions.

From the exact eigenvalues in equation (5.8), the second term satisfies the coercivity estimate

$$\tilde{\text{H}}{|}_{\mathrm{span}{\left(\phi_{0},\phi_{2}\right)}^{⊥}}\leq -3k^{2}I,$$

and the entire stability problem is reduced to understanding the two by two matrix eigenvalue problem defined by $\tilde{\text{H}}{|}_{\mathrm{span}(\phi_{0},\phi_{2})}.$ Furthermore, since the range of $\tilde{\text{H}}$ consists of mean zero functions it follows from the Fredholm alternative that $\tilde{\text{H}}{|}_{\mathrm{span}(\phi_{0},\phi_{2})}$ must have a zero eigenvalue.

This zero eigenvalue is connected with mass conservation. There is a one-parameter family of solutions to this equation of fixed spatial period *L*, which can be thought of as being related to the total mass of the stationary solution. Here we have only considered the simplest solutions—those with zero net mass—but there are analogous expressions in terms of elliptic functions for the general solution. Since we have a one-parameter family of solutions, by Noethers theorem there must be an element in the kernel of linearized operator corresponding to the generator of this family.

It is straightforward to compute the restriction of the linearized operator $\tilde{\text{H}}{|}_{\mathrm{span}(\phi_{0},\phi_{2})}$ in the (independent but non-orthogonal) basis {1,sn^2^(*x*,*k*)} in terms of complete elliptic integrals of the first and the second kind. By using the following identities:

$$\begin{array}{rl} & E(k)=\int_{0}^{1}\frac{\sqrt{1-k^{2}x^{2}}\;dx}{\sqrt{1-x^{2}}},\\ & \int_{-K(k)}^{K(k)}{\mathrm{sn}}^{2}(x,k)\;dx=\frac{2(K(k)-E(k))}{k^{2}}\\ \;\mathrm{and}\; & \int_{-K(k)}^{K(k)}{\mathrm{sn}}^{4}(x,k)\;dx=\frac{\left(4+2k^{2})K(k)-(4+4k^{2})E(k\right)}{3k^{4}}, \end{array}$$

we derive the following expression for $\tilde{\text{H}}{|}_{\mathrm{span}\phi_{0},\phi_{2}}$, the restriction of the operator to the span of the zeroth and second eigenfunctions, in the basis {1,sn^2^(*x*,*k*)}:

$$\tilde{\text{H}}{|}_{\mathrm{span}\phi_{0},\phi_{2}}=\left(\begin{array}{cc} 6\frac{K(k)-E(k)}{K(k)} & \frac{3(1+k^{2})(K(k)-E(k))}{k^{2}K(k)}\\ -6k^{2} & -3(1+k^{2}) \end{array}\right).$$

The eigenvalues of this restriction are given by

5.10 $$\lambda_{0}=0$$

and

5.11 $$\lambda_{1}=\frac{\left(3-3k^{2})K(k)-6E(k\right)}{K(k)}.$$

It is easy to check directly from the definition of the elliptic integrals that the quantity (3−3*k*^2^)*K*(*k*)−6*E*(*k*) is strictly negative for *k*∈[0,1). This follows, for instance, from the Taylor series representations

$$\begin{array}{rl} K(k) & =\frac{\pi }{2}\left(1+\sum_{i=1}^{\infty }{\left(\frac{(2i-1)!!}{(2i)!!}\right)}^{2}k^{2i}\right)\\ E(k) & =\frac{\pi }{2}\left(1-\sum_{i=1}^{\infty }{\left(\frac{(2i-1)!!}{(2i)!!}\right)}^{2}\frac{k^{2i}}{2i-1}\right), \end{array}$$

from which it is easy to see that the quantity (1−*k*^2^)*K*(*k*)−2*E*(*k*) is even with only negative terms in its Taylor series, implying that it is strictly negative. Since the equation conserves mass, it makes sense to consider only perturbations with zero net mass (those orthogonal to the kernel). In this case, we have all eigenvalues strictly in the left half-plane and the stationary solution is nonlinearly stable.

The problem for a general nonlinearity is slightly more complicated, since we do not have explicit formulae for the eigenvalues and eigenfunctions, but it can essentially be completely solved. Assuming that the equation

$$u_{t}=u_{xx}+f(u)-\frac{1}{2L}\int_{-L}^{L}f(u)\;dx,\;u_{x}(\pm L)=0$$

has a stationary solution *u*(*x*), we can compute the linearized operator as

$$\begin{array}{rl} \lambda v & =v_{xx}+f^{\prime }(u)v-\frac{1}{2L}\int_{-L}^{L}f^{\prime }(u)v\;dx,\;v_{x}(\pm L)=0,\\ \Rightarrow \lambda v & =\text{H}v-\frac{1}{2L}\langle \;f^{\prime }(u),v\rangle \text{1}=\tilde{\text{H}}v, \end{array}$$

where **H** is the unperturbed operator: **H***v*=*v*_*xx*_+*f*′(*u*)*v*. It is convenient to introduce a coupling constant *ρ* controlling the strength of the perturbation, although we are mainly interested in the special case *ρ*=1, and we thus consider a one-parameter family of eigenvalue problems

$${\text{H}}_{\rho }v=\text{H}v-\frac{\rho }{2L}\langle \;f^{\prime }(u),v\rangle \text{1}=\lambda v.$$

Applying the Aronszjan–Krein formula gives the following eigenvalue condition:

$$\begin{array}{rl} (\text{H}-\lambda \text{I})v & =\frac{\rho }{2L}\langle \;f^{\prime }(u),v\rangle \text{1}\Rightarrow \\ v & =\frac{\rho }{2L}{\left(\text{H}-\lambda \text{I}\right)}^{-1}\text{1}\langle \;f^{\prime }(u),v\rangle \Rightarrow \\ \left\langle \;f^{\prime }(u),v\right\rangle & =\frac{\rho }{2L}\langle \;f^{\prime }(u),{\left(\text{H}-\lambda \text{I}\right)}^{-1}\text{1}\rangle \langle \;f^{\prime }(u),v\rangle . \end{array}$$

Thus, the spectrum again decomposes into two pieces. Any eigenvectors of **H** which happen to be orthogonal to *f*′(*u*) (〈*f*′(*u*),*v*〉=0) remain eigenvectors of the perturbed problem. Eigenvectors which are not orthogonal to *f*′(*u*) must satisfy the Aronszajn–Krein eigenvalue condition

5.12 $$1=\frac{\rho }{2L}\langle \;f^{\prime }(u),{\left(\text{H}-\lambda \text{I}\right)}^{-1}\text{1}\rangle .$$

(This condition was also identified by Freitas [3], who referred to the eigenvalues which satisfy equation (5.12) as ‘moving eigenvalues’.)

Now note that one has the identity **H1**=*f*′(*u*) and thus we can write

$$\begin{array}{rl} 1 & =\frac{\rho }{2L}\langle \text{H}\text{1},{\left(\text{H}-\lambda \text{I}\right)}^{-1}\text{1}\rangle \\ & =\frac{\rho }{2L}\langle (\text{H}-\lambda \text{I}+\lambda \text{I})\text{1},{\left(\text{H}-\lambda \text{I}\right)}^{-1}\text{1}\rangle \\ & =\rho +\frac{\rho }{2L}\langle \text{1}\lambda ,{\left(\text{H}-\lambda \text{I}\right)}^{-1}\text{1}\rangle \Rightarrow \\ & \Rightarrow \frac{\lambda }{2L}\langle \text{1},{\left(\text{H}-\lambda \text{I}\right)}^{-1}\text{1}\rangle -\frac{1-\rho }{\rho }=0\\ & \Rightarrow \frac{1}{2L}\sum \frac{{\left\langle \text{1},v_{i}\right\rangle }^{2}}{\lambda_{i}-\lambda }-\frac{1-\rho }{\rho \lambda }=h(\lambda )=0, \end{array}$$

where we use the identity $\text{1}=\sum \langle \text{1},v_{i}\rangle v_{i}$ and therefore that ${\left(\text{H}-\lambda \text{I}\right)}^{-1}\text{1}=\sum (\left\langle \text{1},v_{i}\right\rangle /(\lambda_{i}-\lambda v_{i}))$; here, λ_*i*_ and *v*_*i*_ are the eigenvalues and (orthogonal) eigenvectors of the unperturbed operator **H**. Note that in the last expression *h*(λ) is a Herglotz function^4^ —an analytic function that is real on the real axis and maps the open upper half-plane to itself—for *ρ*∈[0,1]. It is well known that the zeroes and poles of a Herglotz function are real, implying that the eigenvalues of the Rubinstein–Sternberg model for *ρ*∈[0,1] are real (see Simon [40] p. 920 for a more detailed discussion of Herglotz functions). This observation is analogous to lemma 5.2 in the paper of Freitas [36], where reality of the eigenvalues for *ρ*∈[0,1] is established using a combination of identities derived from the original equation. In later work, Freitas [10] considers a general rank-one perturbation of a self-adjoint operator: $\tilde{\text{H}}=\text{H}+|a\rangle \langle b|$, and associates with each eigenvalue λ_*i*_ a signature given by sgn(〈*b*,*v*_*i*_〉〈*v*_*i*_,*a*〉), where *v*_*i*_ is the eigenfunction of the unperturbed problem—see, in particular, section 3 of [10]. The condition that all of these signatures are the same is equivalent to requiring that the Aronszajn–Krein function is Herglotz.

This calculation shows that, despite the fact that the linearized operator is not self-adjoint, the spectrum is purely real for *ρ*∈[0,1]. Now let us assume that we understand the spectrum of **H**_0_=**H**, the unperturbed operator, in particular that we know *n*_+_(**H**), the number of positive eigenvalues of the unperturbed operator. Let us consider doing a homotopy in the parameter *ρ*. Since we have shown that the eigenvalues are real the only way that an eigenvalue can move from the right half-plane to the left half-plane (or vice versa) is by passing through the origin. We can detect when this occurs by taking λ=0 in equation (5.12), which becomes

$$1=\frac{\rho }{2L}\langle \;f^{\prime }(u),{\text{H}}^{-1}\text{1}\rangle =\frac{\rho }{2L}\langle \text{1},\text{1}\rangle =\rho .$$

Here we have used the fact that **H1**=*f*′(*u*) so that **1**=**H**^−1^*f*′(*u*). (We are also assuming that **H** is invertible. Minor changes are required in the case that **H** has a kernel; see remark 5.2.) This calculation shows that the unique value of *ρ* for which **H**_*ρ*_ has a zero eigenvalue is *ρ*=1.

We are now in a position to count the number of positive eigenvalues of $\tilde{\text{H}}$ using a continuation argument. The operator $\tilde{\text{H}}$ is bounded above and, by standard arguments, has a finite number of positive eigenvalues. We assume that the number of positive eigenvalues of the unperturbed operator is given by *n*_+_(**H**)=*k*: at *ρ*=0, there are *k* positive eigenvalues and the remaining eigenvalues are negative. For *ρ*∈(0,1), the kernel of **H**_*ρ*_ is empty, so no eigenvalues cross from the left half-line to the right. At *ρ*=1, there is an eigenvalue at λ=0, which either came from the left half-line or the right half-line. We can determine which by computing *dλ*/*dρ* and evaluating at λ=0. Doing so we find that

$$\frac{d\lambda }{d\rho }=-\frac{2L}{\left\langle \;f^{\prime }(u),{\text{H}}^{-2}\text{1}\right\rangle }=-\frac{2L}{\left\langle \text{1},{\text{H}}^{-1}\text{1}\right\rangle }.$$

If 〈**1**,**H**^−1^**1**〉>0, the eigenvalue is moving from the positive half-line to the negative, while if 〈**1**,**H**^−1^**1**〉<0 it is moving from the negative to the positive half-line.

Finally, the assumption that the unperturbed operator **H** is invertible implies that the perturbed operator $\tilde{\text{H}}$ has at most a one-dimensional kernel, since if $\tilde{\text{H}}$ had a higher dimensional kernel one of the eigenfunctions could be chosen to be orthogonal to *f*′(*u*) and would thus lie in the kernel of the unperturbed operator **H**. This completes the proof of the following theorem:

**Theorem 5.1**

*Suppose that the unperturbed operator* $H=\partial_{xx}+f^{\prime }(u)$ *is non-singular and has* $n_{+}(H)=k$*-positive eigenvalues. The perturbed operator* $\tilde{H}$ *has a simple kernel and* $n_{+}(\tilde{H})$*, the number of positive eigenvalues of the linearized operator, is given by*

$$n_{+}(\tilde{H})=\left\{ \begin{array}{ll} k, & \langle 1,H^{-1}1\rangle <0\\ k-1, & \langle 1,H^{-1}1\rangle >0 \end{array}\right.$$

*Thus, a necessary and sufficient condition for spectral stability is that* $n_{+}(H)=0$ *(from which it follows that* $\langle 1,H^{-1}1\rangle <0$*) or* $n_{+}(H)=1$ *and* $\langle 1,H^{-1}1\rangle >0.$

**Remark 5.2**

The case where the unperturbed operator **H** has a kernel is somewhat more involved but can be addressed similarly. There one must do another perturbation calculation near *ρ*=0 to understand how the zero eigenvalue(s) move with *ρ* in order to compute *n*_+_(**H**_*ρ*_) for *ρ* small but non-zero. From there the calculation is the same: the number of positive eigenvalues can stay the same or decrease by one, and this is determined by the sign of 〈**1**,**H**^−1^**1**〉. Here **H** is singular but **1** is in the range of **H**, so ‘**H**^−1^**1**’ may be interpreted in the sense of the Moore–Penrose pseudo-inverse.

As we remarked earlier, there is actually a one-parameter family of stationary solutions to the non-local equation (5.1). We now give an alternative characterization of the stability criterion by expressing it in terms of the dependence of the integrated reaction rate on the mass of the solution.

**Corollary 5.3**

*Two alternative characterizations of the stability criterion in theorem* 5.1 *are as follows*:

1. *Let M denote the total mass of the solution*
  $$M=\int_{-L}^{L}u\;dx$$
  *and R denote the total reaction rate*
  $$R=\int_{-L}^{L}f(u)\;dx.$$
  *Assume that the family of stationary solutions can locally be parametrized by the total mass M, and that the number of positive eigenvalues of the unperturbed operator is given by* $n_{+}(H)=k$. *Then the dimension of the unstable manifold is given by*
  $$n_{+}(\tilde{H})=\left\{ \begin{array}{ll} k, & \frac{dR}{dM}<0\\ k-1, & \frac{dR}{dM}>0. \end{array}\right.$$
  *In particular, a necessary condition for stability is that the total reaction rate must be an increasing function of the total mass*.
2. *Suppose that the stationary solution is given by the quadrature*
  $$\int_{\mu_{-}}^{u}\frac{dy}{\sqrt{2E+2\kappa y-2F(y)}}=x+L,$$
  *for appropriate constants* *E*,*κ*. *Let* *μ*_−_ *and* *μ*_+_ *be two turning points for the quadrature: i.e*. *μ*_±_ *are simple roots of* 2*E*+2*κu*−2*F*(*u*)=0 *such that* 2*E*+2*κu*−2*F*(*u*)>0 *for* *u*∈(*μ*_−_,*μ*_+_), *with* *F* *the antiderivative of the reaction rate*, *F*′(*u*)=*f*(*u*). *Define the period type integrals*
  $$\begin{array}{rl} P(E,\kappa ) & =\frac{1}{2}\oint_{\Gamma }\frac{du}{\sqrt{2E+2\kappa u-2F(u)}},\\ M(E,\kappa ) & =\frac{1}{2}\oint_{\Gamma }\frac{u\;du}{\sqrt{2E+2\kappa u-2F(u)}}\\ \;and\;R(E,\kappa ) & =\frac{1}{2}\oint_{\Gamma }\frac{f(u)\;du}{\sqrt{2E+2\kappa u-2F(u)}}, \end{array}$$
  *where Γ is a simple closed contour containing the branch cut along the real axis from* *μ*_−_ *to* *μ*_+_. *Then the dimension of the unstable manifold is given by*
  $$n_{+}(\tilde{H})=\left\{ \begin{array}{ll} k, & \tau <0\\ k-1, & \tau >0 \end{array}\right.$$
  *where τ is defined by*
  $$\tau =\frac{\left(\partial M/\partial E)(\partial P/\partial \kappa )-(\partial M/\partial \kappa )(\partial P/\partial E\right)}{\left(\partial R/\partial E)(\partial P/\partial \kappa )-(\partial R/\partial \kappa )(\partial P/\partial E\right)},$$
  *and represents the rate of change of M divided by the rate of change of R along the one-parameter family of stationary solutions*.

**Proof.**

The proof consists of computing the family of stationary solutions and observing that translation along the family of stationary solutions generates the appropriate element of the range of **H** needed to compute 〈**1**,**H**^−1^**1**〉.

First, we show that we can find a one-parameter family of stationary solutions through quadrature: see, for instance, the text of Landau & Lifshitz [41]. A stationary solution *u* must satisfy

$$0=u_{xx}+f(u)-\frac{1}{2L}\int_{-L}^{L}f(u)\;dx=u_{xx}+f(u)-\kappa ,$$

for some suitable *κ*; multiplying by *u*_*x*_ we find that

$$0=u_{x}(u_{xx}+f(u)-\kappa )=\frac{d}{dx}\left(\frac{1}{2}u_{x}^{2}+F(u)-\kappa u\right)\Rightarrow \frac{1}{2}u_{x}^{2}+F(u)-\kappa u=E,$$

for some integration constant *E*, and *F*′(*u*)=*f*(*u*). Solving for *u*_*x*_ yields

5.13 $$\frac{du}{\sqrt{2E+2\kappa u-2F(u)}}=dx.$$

The Neumann boundary condition *du*/*dx*=0 is satisfied at a turning point of the function $\sqrt{2E+2\kappa u-2F(u)}$; therefore, we integrate along the real axis between two points *μ*_−_=*u*(−*L*), *μ*_+_=*u*(*L*), at which $\sqrt{2E+2\kappa u-2F(u)}=0$:

5.14 $$\int_{\mu_{-}}^{\mu_{+}}\frac{du}{\sqrt{2E+2\kappa u-2F(u)}}=\int_{-L}^{L}\;dx=2L$$

or

5.15 $$P(E,\kappa )=\frac{1}{2}\oint_{\Gamma }\frac{du}{\sqrt{2E+2\kappa u-2F(u)}}=2L,$$

where *Γ* is a simple closed contour that loops around the segment between *μ*_−_ and *μ*_+_. The factor of $\frac{1}{2}$ in the second equation arises in the usual way, due to the fact that the contributions from the top and the bottom of the square root branch cut add. Condition (5.15) defines a curve in the (*E*,*κ*) plane along which we have a stationary solution, defined locally by the vector field

$$dE\frac{\partial P}{\partial E}+d\kappa \frac{\partial P}{\partial \kappa }=0.$$

If we choose to parametrize the curve (*E*(*s*),*κ*(*s*)) in the (*E*,*κ*) plane by arc length *s*, then we can take

5.16 $$\frac{dE}{ds}=-\frac{P_{\kappa }}{\sqrt{P_{\kappa }^{2}+P_{E}^{2}}}$$

and

5.17 $$\frac{d\kappa }{ds}=\frac{P_{E}}{\sqrt{P_{\kappa }^{2}+P_{E}^{2}}}.$$

Note that along this curve, we have the identities

$$\begin{array}{rl} R(E(s),\kappa (s)) & =\frac{1}{2}\oint_{\Gamma }\frac{f(u)\;du}{\sqrt{2E+2\kappa u-2F(u)}}=\int_{-L}^{L}f(u(x;s))\;dx,\\ M(E(s),\kappa (s)) & =\frac{1}{2}\oint_{\Gamma }\frac{u\;du}{\sqrt{2E+2\kappa u-2F(u)}}=\int_{-L}^{L}u(x;s)\;dx\\ \;\mathrm{and}\;P(E(s),\kappa (s)) & =2L. \end{array}$$

Also note that one has the identity *κP*(*E*,*κ*)=*R*(*E*,*κ*), so one could in principle eliminate one of these quantities although we have chosen not to do so here.

Having found a family of stationary solutions parametrized by the arc length, *u*(*x*;*s*), we now proceed to compute the quantity 〈**1**,**H**^−1^**1**〉 in terms of *M* and *R*. First, take the equation for the stationary solution

$$u_{xx}(x;s)+f(u(x;s))-\frac{1}{2L}\int_{-L}^{L}f(u(x;s))\;dx=0,$$

and differentiate with respect to the arc length parameter *s*, giving

5.18 $$u_{sxx}+f^{\prime }(u)u_{s}-\frac{1}{2L}\int_{-L}^{L}f^{\prime }(u)u_{s}\;dx=0,$$

which we recognize as (recalling that **H***v*=*v*_*xx*_+*f*′(*u*)*v*)

$$\text{H}u_{s}=\frac{1}{2L}\frac{dR}{ds}.$$

The right-hand side is a constant, thus we have

$$u_{s}=\left(\frac{1}{2L}\frac{dR}{ds}\right){\text{H}}^{-1}\text{1}.$$

Integrating this identity gives

5.19 $$\frac{1}{2L}\frac{dR}{ds}\langle \text{1},{\text{H}}^{-1}\text{1}\rangle =\langle \text{1},u_{s}\rangle =\int_{-L}^{L}u_{s}\;dx=\frac{dM}{ds}$$

or

5.20 $$\langle \text{1},{\text{H}}^{-1}\text{1}\rangle =2L\frac{dM/ds}{dR/ds}.$$

Since *L*>0 is positive, the quantity 〈**1**,**H**^−1^**1**〉 is positive if *R* increases with increasing *M* and is negative if *R* decreases with increasing *M*. Applying the chain rule and equations (5.16) and (5.17) we find that

5.21 $$\begin{array}{rl} \left\langle \text{1},{\text{H}}^{-1}\text{1}\right\rangle & =2L\frac{\left(\partial M/\partial E)(dE/ds)+(\partial M/\partial \kappa )(d\kappa /ds\right)}{\left(\partial R/\partial E)(dE/ds)+(\partial R/\partial \kappa )(d\kappa /ds\right)}\\ & =2L\frac{\left(\partial M/\partial E)(\partial P/\partial \kappa )-(\partial M/\partial \kappa )(\partial P/\partial E\right)}{\left(\partial R/\partial E)(\partial P/\partial \kappa )-(\partial R/\partial \kappa )(\partial P/\partial E\right)}=2L\tau . \end{array}$$

 □

While we are not aware of previous results of this type for dissipative equations, there are a number of results for the stability of nonlinear dispersive waves that relate the index of some linearized operator to the sign of the derivative of some conserved quantity with respect to a parameter, analogous to the result presented here. The classical Vakhitov–Kolokolov criteria, relating the stability of solitary wave solutions *ψ*(*x*,*t*)=*e*^*iωt*^*ϕ*(*x*;*ω*) to the nonlinear Schrödinger equation

$$i\psi_{i}=-\psi_{xx}+g(|\psi {|}^{2})\psi ,$$

to the sign of $(d/d\omega )\int |\phi {|}^{2}(x;\omega )\;dx$ falls into this category [42–45]. The problem of the stability of periodic solutions to nonlinear dispersive waves is perhaps even more similar to the present case: in this situation, the index is determined by the signs of certain determinants of derivatives of period integrals (see [46–48], for examples).

It is worth discussing the relationship of this result to the results of Freitas [36], who relates the dimension of the unstable manifold to the lap number of the underlying solution. Specifically Freitas shows (see Theorems 5.1 and 5.13 of [36]) that the dimension of the unstable manifold is related to *m*, the number of extrema of the underlying solution *u* in (−*L*,*L*) via

$$m\leq n_{+}(\tilde{\text{H}})\leq m+2.$$

Thus, the dimension of the unstable manifold can differ from the number of extrema by up to two. To summarize the reasoning, the unstable manifold of the unperturbed operator has dimension equal to the lap number, *l*(*w*)=*m*+1; the corresponding dimension for the perturbed operator can then go up (to *m*+2) or down (to *m*) by at most one.

Here, we determine this direction (whether up to *m*+2, or down to *m*), by computing the index of the unperturbed operator; in terms of *m*, our result states that

$$\begin{array}{ll} m\leq n_{+}(\tilde{\text{H}})\leq m+1, & \;\mathrm{if}\;\langle \text{1},{\text{H}}^{-1}\text{1}\rangle >0\\ m+1\leq n_{+}(\tilde{\text{H}})\leq m+2 & \;\mathrm{if}\;\langle \text{1},{\text{H}}^{-1}\text{1}\rangle <0, \end{array}$$

where the sign of 〈**1**,**H**^−1^**1**〉 can be replaced by the sign of *dR*/*dM* or any of the other equivalent forms discussed previously.

## 6. Discussion

In conclusion, we have presented a general method of analysing low-rank perturbations of self-adjoint operators. We show how to use a simple idea of classical differential geometry (the envelope of a family of curves) to completely analyse the spectrum. When the rank of the perturbation is two, this allows us to view the system in a geometric way through a phase diagram in the perturbation strengths (*ρ*_1_,*ρ*_2_). By locating constant eigenvalue and eigenvalue coincidence curves (both computable through simple formulas), we can determine where the perturbed operator is stable, and where double real eigenvalues bifurcate into complex pairs. This latter situation (bifurcation into a complex pair) coincides with a poorly conditioned eigenvalue, which in turn signals that small changes in the perturbation parameter will yield large changes in the operator behaviour.

We used these techniques to analyse three problems of this form; a model of the oculomotor integrator due to Anastasio & Gad [1], a continuum version of the oculomotor integrator model, and a non-local model of phase separation due to Rubinstein & Sternberg [2]. In the first two problems, the physical interpretation of our model (a neural network that must maintain a steady eye position in the absence of input) required that we identify (a) where the perturbed system had a specific eigenvalue and (b) where this particular eigenvalue would be poorly conditioned. Our results in §2 show that both (a) and (b) can only occur in proximity to a specific point on the (*ρ*_1_,*ρ*_2_) plane, which was then easy to visualize. In §§3 and 4, some portions of the model are not completely specified by biology (such as the vestibular- to-Purkinje connections), but must be chosen arbitrarily (even randomly). In this paper, we analysed a few carefully chosen examples. But, the geometric method we describe here also gives us a rapid way to survey a large family of such models; using such a survey to draw conclusions about the vestibular-to-cerebellar pathway is an area for future work.

The problem analysed in §5 involves a rank one (rather than rank two) perturbation, and so a phase plane approach is not applicable. Instead, we systematically exploit the rank-one nature of the perturbation to characterize stability of stationary solutions in terms of the unperturbed operator. We further show how to construct a one-parameter family of stationary solutions, and relate the stability condition to the relative change in two integrated quantities (mass and reaction rate) as one travels along this family.

In analysing these three problems, we have by no means exhausted the possible applications. For example, the eigenvalue problem in equation (4.3) is very similar to the stability problem for spike solutions to activator–inhibitor models in the limit of slow activator diffusion [3,4] (although the problem we study here differs because the eigenvalue enters in a nonlinear way). Similar models of reaction–diffusion equations with non-local interactions have arisen in a number of other contexts including population dynamics [49], runaway ohmic heating [5–7] and microwave heating [8]. Therefore, we anticipate that the techniques presented here should be applicable to understanding these problems.

## Footnotes

1 For purposes of exposition, we will consider the case where $\tilde{\text{M}}$ is a matrix, but everything we say will apply equally to the case where $\tilde{\text{M}}$ is an operator of compact resolvent.

2 We have also scaled the entire problem by the common factor of *α*.

3 For fixed period *L*, there is actually a one-parameter family of front solutions. The one given here has zero net mass and is the simplest. The rest will be discussed later.

4 Sometimes called a Nevanlinna or Nevanlinna–Pick function.

## Data accessibility

Not applicable: the paper contains sufficient detail to reproduce the results.

## Appendix A

**A.1. Derivation of equations (2.2) and (2.3)**

Here we include details for the derivation of equation (2.2), for matrices. We will make repeated use of the matrix determinant lemma:

A 1 $$\det(\text{A}+uv^{t})=(1+v^{t}{\text{A}}^{-1}u)\det\text{A},$$

and the Sherman–Morrison formula:

A 2 $${\left(\text{A}+uv^{t}\right)}^{-1}={\text{A}}^{-1}-\frac{{\text{A}}^{-1}uv^{t}{\text{A}}^{-1}}{1+v^{t}{\text{A}}^{-1}u}.$$

We first apply equation (A 1) with $\text{A}=\text{M}-\lambda \text{I}+\rho_{1}f_{1}g_{1}^{t}$, ***u***=*ρ*_2_***f***_2_, and ***v***=***g***_2_, resulting in equation (A 3). We then apply Sherman–Morrison to the inverse matrix, resulting in equation (A 4). Finally, we apply equation (A 1) a second time, but with **A**=**M**−λ**I**, ***u***=*ρ*_1_***f***_1_ and ***v***=***g***_1_, and simplify to emphasize the polynomial form in *ρ*_1_,*ρ*_2_:

A 3 $$\begin{array}{rl} & \det(\tilde{\text{M}}-\lambda \text{I})=\det(\text{M}-\lambda \text{I}+\rho_{1}f_{1}{g_{1}}^{t}+\rho_{2}f_{2}g_{2}^{t})\\ & \;=(1+\rho_{2}g_{2}^{t}{\left(\text{M}-\lambda \text{I}+\rho_{1}f_{1}g_{1}^{t}\right)}^{-1}f_{2})\times \det(\text{M}-\lambda \text{I}+\rho_{1}f_{1}g_{1}^{t}) \end{array}$$

A 4 $$\begin{array}{rl} & \;=\left(1+\rho_{2}g_{2}^{t}\left({\left(\text{M}-\lambda \text{I}\right)}^{-1}-\frac{{\left(\text{M}-\lambda \text{I}\right)}^{-1}\rho_{1}f_{1}g_{1}^{t}{\left(\text{M}-\lambda \text{I}\right)}^{-1}}{1+g_{1}^{t}{\left(\text{M}-\lambda \text{I}\right)}^{-1}\rho_{1}f_{1}}\right)f_{2}\right)\times \det(\text{M}-\lambda \text{I}+\rho_{1}f_{1}g_{1}^{t}) \end{array}$$

A 5 $$\begin{array}{rl} & \;=\left(1+\rho_{2}g_{2}^{t}\left({\left(\text{M}-\lambda \text{I}\right)}^{-1}-\frac{{\left(\text{M}-\lambda \text{I}\right)}^{-1}\rho_{1}f_{1}g_{1}^{t}{\left(\text{M}-\lambda \text{I}\right)}^{-1}}{1+g_{1}^{t}{\left(\text{M}-\lambda \text{I}\right)}^{-1}\rho_{1}f_{1}}\right)f_{2}\right)\\ & \;\times (1+g_{1}^{t}{\left(\text{M}-\lambda \text{I}\right)}^{-1}\rho_{1}f_{1})\times \det(\text{M}-\lambda \text{I}) \end{array}$$

A 6 $$\begin{array}{rl} & \;=\det(\text{M}-\lambda \text{I})\times [1+\rho_{1}g_{1}^{t}{\left(\text{M}-\lambda \text{I}\right)}^{-1}f_{1}+\rho_{2}g_{2}^{t}{\left(\text{M}-\lambda \text{I}\right)}^{-1}f_{2}\\ & \;+\rho_{1}\rho_{2}((g_{1}^{t}{\left(\text{M}-\lambda \text{I}\right)}^{-1}f_{1})(g_{2}^{t}{\left(\text{M}-\lambda \text{I}\right)}^{-1}f_{2})-(g_{2}^{t}{\left(\text{M}-\lambda \text{I}\right)}^{-1}f_{1})(g_{1}^{t}{\left(\text{M}-\lambda \text{I}\right)}^{-1}f_{2}))]. \end{array}$$

Using the cofactor formula ${\mathrm{cof}}^{t}\text{A}=\det(\text{A}){\text{A}}^{-1}$ to replace each instance of $\det(\text{M}-\lambda \text{I}){\left(\text{M}-\lambda \text{I}\right)}^{-1}$ with cof^*t*^(**M**−λ**I**) gives the formula in equation (2.2).

To derive equation (2.3), use the spectral decomposition for self-adjoint operators:

$$\text{M}-\lambda \text{I}=\sum_{i}(\lambda_{i}-\lambda )\phi_{i}\phi_{i}^{t},$$

where λ_*i*_ and ***ϕ***_*i*_ are the eigenvalue and eigenvectors of the unperturbed operator, respectively; substitute into equation (A 6) and use the orthogonality of eigenfunctions ($\phi_{i}^{t}\phi_{j}=0$ for *i*≠*j*).

**A.2. Derivation of equation (2.4)**

We fill in a few more details for calculating eigenvalues of:

$$\tilde{\text{M}}w=\text{M}w+\rho_{1}f_{1}\langle g_{1},w\rangle +\rho_{2}f_{2}\langle g_{2},w\rangle =\lambda w,$$

where $\tilde{\text{M}}$ is an operator with compact resolvent.

First, act on this by the resolvent of the unperturbed operator, *R*_λ_=(**M**−λ**I**)^−1^:

A 7 $$\begin{array}{rl} 0 & =(\text{M}-\lambda \text{I})w+\rho_{1}f_{1}\langle g_{1},w\rangle +\rho_{2}f_{2}\langle g_{2},w\rangle \Rightarrow \end{array}$$

A 8 $$\begin{array}{rl} R_{\lambda }0 & =R_{\lambda }(\text{M}-\lambda \text{I})w+\rho_{1}R_{\lambda }f_{1}\langle g_{1},w\rangle +\rho_{2}R_{\lambda }f_{2}\langle g_{2},w\rangle \end{array}$$

A 9 $$\begin{array}{rl} 0 & =w+\rho_{1}R_{\lambda }f_{1}\langle g_{1},w\rangle +\rho_{2}R_{\lambda }f_{2}\langle g_{2},w\rangle \Rightarrow \end{array}$$

A 10 $$\begin{array}{rl} \;\text{and}\;-w & =\rho_{1}R_{\lambda }f_{1}\langle g_{1},w\rangle +\rho_{2}R_{\lambda }f_{2}\langle g_{2},w\rangle . \end{array}$$

Now, act on the equation by both ***g***_1_ and ***g***_2_, to yield two consistency conditions:

A 11 $$\begin{array}{rl} -\langle g_{1},w\rangle & =\rho_{1}\langle g_{1},R_{\lambda }f_{1}\rangle \langle g_{1},w\rangle +\rho_{2}\langle g_{1},R_{\lambda }f_{2}\rangle \langle g_{2},w\rangle \end{array}$$

A 12 $$\begin{array}{rl} -\langle g_{2},w\rangle & =\rho_{1}\langle g_{2},R_{\lambda }f_{1}\rangle \langle g_{1},w\rangle +\rho_{2}\langle g_{2},R_{\lambda }f_{2}\rangle \langle g_{2},w\rangle \Rightarrow \end{array}$$

A 13 $$\begin{array}{rl} \;\text{and therefore}\;\langle g_{2},w\rangle & =\frac{-\rho_{1}\langle g_{2},R_{\lambda }f_{1}\rangle \langle g_{1},w\rangle }{1+\rho_{2}\langle g_{2},R_{\lambda }f_{2}\rangle }. \end{array}$$

Substituting equation (A 13) into equation (A 11) and dividing by the now common factor 〈***g***_1_,***w***〉, will yield a single polynomial equation for *ρ*_1_ and *ρ*_2_:

A 14 $$0=1+\rho_{1}\langle g_{1},R_{\lambda }f_{1}\rangle +\rho_{2}\langle g_{2},R_{\lambda }f_{2}\rangle +\rho_{1}\rho_{2}(\langle g_{1},R_{\lambda }f_{1}\rangle \langle g_{2},R_{\lambda }f_{2}\rangle -\langle g_{1},R_{\lambda }f_{2}\rangle \langle g_{2},R_{\lambda }f_{1}\rangle ).$$

When ***g***_1_=***g***_2_, the final *ρ*_1_*ρ*_2_ term is zero; this is what happens in the continuum example presented in §3.

We now explain how to apply this formalism to the eigenvalue problem in §3:

A 15 $$\beta {\left(\Delta x\right)}^{2}\psi_{xx}+(-1+3\beta )\psi +\frac{\rho_{1}\langle \psi ,1\rangle }{\Delta x(\lambda +1)}\delta (x-x_{1})+\frac{\rho_{2}\langle \psi ,1\rangle }{\Delta x(\lambda +1)}\delta (x-x_{2})=\lambda \psi ,\;\psi (0)=0=\psi (L).$$

Here, **M**=*β*(*Δx*)^2^*ψ*_*xx*_+(−1+3*β*)*ψ* and (as we expect negative eigenvalues) we will act on equation (A.15) with the resolvent operator *R*_λ_≡(*β*(*Δx*)^2^*ψ*_*xx*_+(−1+3*β*)*ψ*−*λψ*)^−1^. Here ***f***_1_∝*δ*(*x*−*x*_1_) and ***f***_2_∝*δ*(*x*−*x*_2_); therefore, *R*_λ_***f***_1_ is equivalent to solving Green’s function problem

$$\beta {\left(\Delta x\right)}^{2}\psi_{xx}+(-1+3\beta )\psi -\lambda \psi =\delta (x-x_{1});\;\psi (0)=\psi (1)=0.$$

Since *ψ* solves the PDE *ψ*_*xx*_+((−1−λ+3*β*)/*βΔx*^2^)*ψ*=0 for any *x*≠*x*_1_,*x*_2_, *ψ* must have the following form:

A 16 $$\psi (x)=\left\{ \begin{array}{ll} A\;\sin\;\omega x, & x<x_{1}\\ B\;\sin\;\omega x+C\;\cos\;\omega x, & x_{1}<x<x_{2}\\ D\;\sin\;(\omega (L-x)), & x>x_{2} \end{array}\right.$$

where *ω*^2^=−1−λ+3*β*/*β*(*Δx*)^2^. By linearity of the resolvent, and the fact that ***g***_1_=***g***_2_, we can solve them together: i.e. *ρ*_1_〈***g***_1_,*R*_λ_***f***_1_〉+*ρ*_2_〈***g***_2_,*R*_λ_***f***_2_〉=*ρ*_1_〈***g***_1_,*R*_λ_***f***_1_〉+*ρ*_2_〈***g***_1_,*R*_λ_***f***_2_〉=〈***g***_1_,*ρ*_1_*R*_λ_***f***_1_+*ρ*_2_*R*_λ_***f***_2_〉=〈***g***_1_,*R*_λ_(*ρ*_1_***f***_1_+*ρ*_2_***f***_2_)〉.

Therefore, we solve Green’s function problem only once; *ψ* must satisfy the following conditions, which impose continuity of *ψ* at *x*_1_,*x*_2_ and the appropriate jump of the derivatives:

A 17 $$\begin{array}{rl} \psi^{+}(x_{1}) & =\psi^{-}(x_{1}), \end{array}$$

18 $$\begin{array}{rl} \psi^{+}(x_{2}) & =\psi^{-}(x_{2}), \end{array}$$

A 19 $$\begin{array}{rl} \psi_{x}^{+}(x_{1})-\psi_{x}^{-}(x_{1}) & =-\frac{\rho_{1}}{\beta {\left(\Delta x\right)}^{3}(\lambda +1)}=-\frac{\rho_{1}}{\beta^{2}{\left(\Delta x\right)}^{3}(3-{\left(\Delta x\right)}^{2}\omega^{2})} \end{array}$$

A 20 $$\begin{array}{rl} \;\text{and}\;\psi_{x}^{+}(x_{2})-\psi_{x}^{-}(x_{2}) & =-\frac{\rho_{2}}{\beta {\left(\Delta x\right)}^{3}(\lambda +1)}=-\frac{\rho_{2}}{\beta^{2}{\left(\Delta x\right)}^{3}(3-{\left(\Delta x\right)}^{2}\omega^{2})}. \end{array}$$

This yields a system of four equations in the four unknowns *A*, *B*, *C* and *D*. Similarly, we can express the action of ***g***_1_ on any function of the form equation (A.16) as a vector inner product: in this case,

A 21 $$\langle 1,\psi \rangle =g^{T}w$$

and

A 22 $$g=\frac{1}{\omega }\left[\begin{array}{c} 1-\cos\;\omega x_{1}\\ \cos\;\omega x_{1}-\cos\;\omega x_{2}\\ \sin\;\omega x_{2}-\sin\;\omega x_{1}\\ 1-\cos\;(\omega (L-x_{2})) \end{array}\right];\;w=\left[\begin{array}{c} A\\ B\\ C\\ D \end{array}\right].$$

Thus, our final polynomial is $0=1+g^{T}w$, where w is the vector of coefficients we obtained by solving equations (A 17)–(A 20).

## References

[1] Anastasio TJ, Gad YP 2007 Sparse cerebellar innervation can morph the dynamics of a model oculomotor neural integrator. *J. Comput. Neurosci.* 22, 239–254. (doi:10.1007/s10827-006-0010-x) 17086435 10.1007/s10827-006-0010-x

[2] Rubinstein J, Sternberg P 1992 Nonlocal reaction-diffusion equations and nucleation. *IMA J. Appl. Math.* 48, 249–264. (doi:10.1093/imamat/48.3.249)

[3] Freitas P 1994 A nonlocal Sturm-Liouville eigenvalue problem. *Proc. R. Soc. Edinburgh* 124A, 169–188. (doi:10.1017/S0308210500029279)

[4] Iron D, Ward MJ 2002 The dynamics of multispike solutions to the one-dimensional Gierer-Meinhardt model. *SIAM J. Appl. Math.* 62, 1924–1951. (electronic) (doi:10.1137/S0036139901393676)

[5] Chafee N 1981 The electric ballast resistor: homogeneous and nonhomogeneous equilibria. In *Nonlinear differential equations (Proc. Internat. Conf., Trento, 1980)* (eds P de Mottoni, L Salvadori), pp. 97–127. New York, NY: Academic Press.

[6] Lacey AA 1995 Thermal runaway in a non-local problem modelling Ohmic heating. I. Model derivation and some special cases. *Eur. J. Appl. Math.* 6, 127–144. (doi:10.1017/S095679250000173X)

[7] Lacey AA 1995 Thermal runaway in a non-local problem modelling Ohmic heating. II. General proof of blow-up and asymptotics of runaway. *Eur. J. Appl. Math.* 6, 201–224. (doi:10.1017/S0956792500001807)

[8] Bose A, Kriegsmann GA 1998 Stability of localized structures in non-local reaction-diffusion equations. *Methods Appl. Anal.* 5, 351–366. (doi:10.4310/MAA.1998.v5.n4.a2)

[9] Du Y, Hsu SB 2010 On a nonlocal reaction-diffusion problem arising from the modeling of phytoplankton growth. *SIAM J. Math. Anal.* 42, 1305–1333. (doi:10.1137/090775105)

[10] Freitas P 1999 Nonlocal reaction diffusion equations. *Fields Inst. Commun.* 21, 187–204.

[11] Bruce JW, Giblin PJ 1984 *Curves and singularities*. Cambridge, UK: Cambridge University Press.

[12] Spivak M 1999 *Differential geometry*, vol. III, 3rd edn Berkeley, MA: Publish or Perish.

[13] Robinson DA 1989 Control of eye movements. In *Handbook of physiology, section 1: the nervous system*, (ed. VB Brooks), vol. 2, part 2, pp. 1275–1320. Bethesda, MD: American Physiological Society.

[14] Robinson DA 1989 Integrating with neurons. *Annu. Rev. Neurosci.* 12, 33–45. (doi:10.1146/annurev.ne.12.030189.000341) 2648952 10.1146/annurev.ne.12.030189.000341

[15] Barreiro AK, Bronski JC, Anastasio TJ 2009 Bifurcation theory explains waveform variability in a congenital eye movement disorder. *J. Comput. Neurosci.* 26, 321–329. (doi:10.1007/s10827-008-0113-7) 18758933 10.1007/s10827-008-0113-7

[16] Barreiro AK 2012 Mechanisms of neural integration: recent results and relevance to nystagmus modeling. In *The challenge of nystagmus* (eds CM Harris, I Gottlob, J Sanders), pp. 75–90. Proceedings of the Second International Research Workshop on Nystagmus 2009. Cardiff, UK: UK Nystagmus Network.

[17] Berthoz A, Jones GM 1985 *Adaptive mechanisms in gaze control: facts and theories*. Amsterdam, The Netherlands: Elsevier.

[18] Gonshor A, Jones JGM 1976 Short-term adaptive changes in the human vestibulo-ocular reflex arc. *J. Physiol.* 256, 361–379. (doi:10.1113/jphysiol.1976.sp011329) 16992507 10.1113/jphysiol.1976.sp011329 PMC1309312

[19] Gonshor A, Jones JGM 1976 Extreme vestibulo-ocular adaptation induced by prolonged optical reversal of vision. *J. Physiol.* 256, 381–414. (doi:10.1113/jphysiol.1976.sp011330) 16992508 10.1113/jphysiol.1976.sp011330 PMC1309313

[20] Tiliket C, Shelhamer M, Roberts D, Zee DS 1994 Short term vestibulo-ocular reflex adaptation in humans. I. Effect on the ocular motor velocity-to-position neural integrator. *Exp. Brain Res.* 100, 316–327. (doi:10.1007/BF00227201) 7813668 10.1007/BF00227201

[21] Robinson D 1974 The effect of cerebellectomy on the cat’s vestibuloocular integrator. *Brain Res.* 71, 195–207. (doi:10.1016/0006-8993(74)90961-5) 4468058 10.1016/0006-8993(74)90961-5

[22] Robinson DA 1976 Adaptive gain control of vestibuloocular reflex by the cerebellum. *J. Neurophysiol.* 39, 954–969. 1086347 10.1152/jn.1976.39.5.954

[23] Büttner-Ennever JA 1988 *Neuroanatomy of the oculomotor system*. Amsterdam, The Netherlands: Elsevier.

[24] Zee S, Yamazaki A, Butler PH, Gücer G 1981 Effects of ablation of flocculus and paraflocculus on eye movements in primate. *J. Neurophysiol.* 46, 878–899. 7288469 10.1152/jn.1981.46.4.878

[25] Chelazzi L, Ghirardi M, Rossi F, Strata P, Tempia F 1990 Spontaneous saccades and gaze holding ability in the pigmented rat. II. Effects of localized cerebellar lesions. *Eur. J. Neurosci.* 2, 1085–1094. (doi:10.1111/j.1460-9568.1990.tb00020.x) 12106069 10.1111/j.1460-9568.1990.tb00020.x

[26] Rambold H, Churchland A, Selig Y, Jasmin L, Lisberger SG 2002 Partial ablations of the flocculus and ventral paraflocculus in monkeys cause linked deficits in smooth pursuit eye movements and adaptive modification of the VOR. *J. Neurophysiol.* 87, 912–924. (doi:10.1152/jn.00768.2000) 11826056 10.1152/jn.00768.2000 PMC2629758

[27] Nagao S, Kitazawa H 2003 Effects of reversible shutdown of the monkey flocculus on the retention of adaptation of the horizontal vestibulo-ocular reflex. *Neuroscience* 118, 954–969. (doi:10.1016/S0306-4522(02)00991-0) 10.1016/s0306-4522(02)00991-0 12699790

[28] Epema AH, Gerrits NM, Voogd J 1990 Secondary vestibulocerebellar projections to the flocculus and uvulo-nodular lobule of the rabbit: a study using HRP and double fluorescent tracer techniques. *Exp. Brain Res.* 80, 72–82. (doi:10.1007/BF00228849) 2358039 10.1007/BF00228849

[29] Langer T, Fuchs AF, Scudder CA, Chubb MC 1985 Afferents to the flocculus of the cerebellum in the rhesus macaque as revealed by retrograde transport of horseradish peroxidase. *J. Comp. Neurol.* 235, 1–25. (doi:10.1002/cne.902350102) 3989000 10.1002/cne.902350102

[30] Langer T, Fuchs AF, Chubb MC, Scudder CA, Lisberger SG 1985 Floccular efferents in the rhesus macaque as revealed by autoradiography and horseradish peroxidase. *J. Comp. Neurol.* 235, 26–37. (doi:10.1002/cne.902350103) 3989003 10.1002/cne.902350103

[31] Sekirnjak C, Vissel B, Bollinger J, Faulstich M 2003 Purkinje cell synapses target physiologically unique brainstem neurons. *J. Neurosci.* 23, 6392–6398. 12867525 10.1523/JNEUROSCI.23-15-06392.2003 PMC6740533

[32] Tan H, Gerrits NM 1992 Laterality in the vestibulo-cerebellar mossy fiber projection to flocculus and caudal vermis in the rabbit: A retrograde fluorescent double-labeling study. *Neuroscience* 47, 909–919. (doi:10.1016/0306-4522(92)90039-5) 1374542 10.1016/0306-4522(92)90039-5

[33] Babalian AL, Vidal PP 2000 Floccular modulation of vestibuloocular pathways and cerebellum-related plasticity: an *in vitro* whole brain study. *J. Neurophysiol.* 84, 2514–2528. 11067994 10.1152/jn.2000.84.5.2514

[34] Keener JP 1988 *Principles of applied mathematics: transformation and approximation*. Advanced Book Program Reading, MA: Addison Wesley.

[35] Freitas P 1994 Bifurcation and stability of stationary solutions of nonlocal scalar reaction-diffusion equations. *J. Dyn. Diff. Equ.* 6, 613–629. (doi:10.1007/BF02218850)

[36] Freitas P 1995 Stability for stationary solutions for a scalar nonlocal reaction diffusion equation. *Q. J. Mech. Appl. Math.* 48, 557–582. (doi:10.1093/qjmam/48.4.557)

[37] Bates PW, Fife PC 1993 Spectral comparison principles for the Cahn-Hilliard and phase field equations, and time scales for coarsening. *Phys. D* 53, 990–1008.

[38] Magnus W, Winkler S 1966 *Hill’s equation*. Interscience Tracts in Pure and Applied Mathematics, no. 20 New York, NY: Interscience Publishers John Wiley & Sons. (MathSciNet MR0197830)

[39] Eastham MSP 1973 *The spectral theory of periodic differential equations*. Texts in Mathematics (Edinburgh) New York, NY: Hafner Press. (MathSciNet MR3075381)

[40] Simon B 2005 Orthogonal polynomials on the unit circle. Part 2: spectral theory. In *American Mathematical Society Colloquium Publications*, vol. 54, pp. 467–1044. Providence, RI: American Mathematical Society.

[41] Landau LD, Lifshitz EM 1976 *Course of theoretical physics*, vol. 1, 3rd edn Oxford, NY: Pergamon Press Mechanics, Translated from the Russian by JB Skyes and JS Bell.

[42] Vakhitov NG, Kolokolov AA 1973 Stationary solutions of the wave equation in a medium with nonlinearity saturation. *Radiophys. Quantum Electron.* 16, 783–789. (doi:10.1007/BF01031343)

[43] Grillakis M, Shatah J, Strauss W 1987 Stability theory of solitary waves in the presence of symmetry. I. *J. Funct. Anal.* 74, 160–197. (doi:10.1016/0022-1236(87)90044-9)

[44] Grillakis M, Shatah J, Strauss W 1990 Stability theory of solitary waves in the presence of symmetry. II. *J. Funct. Anal.* 94, 308–348. (doi:10.1016/0022-1236(90)90016-E)

[45] Pego RL, Weinstein MI 1992 Eigenvalues, and instabilities of solitary waves. *Phil. Trans. R. Soc. Lond. A* 340, 47–94. (doi:10.1098/rsta.1992.0055)

[46] Kapitula T, Deconinck B 2015 On the spectral and orbital stability of spatially periodic stationary solutions of generalized Korteweg–de Vries equations. In *Hamiltonian partial differential equations and applications*. Fields Institute Communications, vol. 75 (eds P Guyenne, D Nicholls, C Sulem), pp. 285–322. Toronto, ON: Fields Institute for Research in Mathematical Sciences.

[47] Bronski JC, Johnson MA 2010 The modulational instability for a generalized Korteweg-de Vries equation. *Arch. Ration. Mech. Anal.* 197, 357–400. (doi:10.1007/s00205-009-0270-5)

[48] Bronski JC, Johnson MA, Kapitula T 2011 An index theorem for the stability of periodic travelling waves of Korteweg-de Vries type. *Proc. R. Soc. Edinb. A* 141, 1141–1173. (doi:10.1017/S0308210510001216)

[49] Furter J, Grinfeld M 1989 Local vs. nonlocal interactions in population dynamics. *J. Math. Biol.* 27, 65–80. (doi:10.1007/BF00276081)

